# Vector competence of temperate and tropical mosquito species for Babanki virus

**DOI:** 10.1371/journal.pntd.0013051

**Published:** 2026-08-24

**Authors:** Mathilde Ban, Virginie Geolier, Emeline Perthame, Elisabeth Ferquel, Martin Faye, Moussa Moise Diagne, El Hadji Ndiaye, Gamou Fall, Mawlouth Diallo, Dimitri Lavillette, Valérie Choumet

**Affiliations:** 1 Global Health Department, Environment and Infectious Risk Unit, Institut Pasteur, Université Paris Cité, Paris, France; 2 Bioinformatic and biostatistics Hub, Institut Pasteur, Université Paris Cité, Paris, France; 3 Virology Unit, Institut Pasteur de Dakar, Dakar, Senegal; 4 Medical Zoology Unit, Institut Pasteur de Dakar, Dakar, Senegal; 5 International Infectiology Research Center, HepVir, Inserm U1111-Université Claude Bernard Lyon 1, CNRS, UMR5308, Ecole Normale Supérieure de Lyon, Université Lyon, Lyon, France; Faculty of Science, Ain Shams University (ASU), EGYPT

## Abstract

Babanki virus (BBKV), an alphavirus belonging to the Western Equine Encephalitis antigenic complex, was first isolated from *Mansonia africana* mosquitoes in 1969 but remains poorly characterized. Transmitted to humans and animals through the bite of an infectious female mosquito, it is associated with febrile illness, rash, and arthritis, similarly to its close relative Sindbis virus. No specific treatment or vaccine is currently available. Here, we experimentally assessed the vector competence of mosquito species from tropical and temperate regions under ecologically relevant temperature conditions. Using a BBKV molecular clone, individual midguts, salivary glands, legs, and saliva samples were collected at different time points to assess infection, dissemination, and transmission dynamics in *Aedes* (*Aedes albopictus* from Nice and Réunion Island, and *Aedes aegypti*) and *Culex* (*Culex pipiens molestus* from Paris and *Culex quinquefasciatus*) mosquitoes. We show that BBKV efficiently infects and disseminates in all tested species, with *Aedes* mosquitoes exhibiting higher infection rates (>70%) compared to *Culex* species. Importantly, infectious viral particles were detected in mosquito saliva, confirming transmission potential. Viral replication persisted over time and was supported by both RNA quantification and infectious virus titration. Overall, our results show that multiple mosquito species from both temperate and tropical regions are competent vectors for BBKV, highlighting a significant emergence potential.

## Author summary

Babanki virus (BBKV) is a non-characterized arbovirus identified in 1969 in Cameroon. BBKV is closely related to Sindbis virus which causes symptoms like febrile illness, rash, and arthritis in humans and animals. Both are alphaviruses within the Western Equine Encephalitis complex. BBKV is identified only in the African continent so far. Despite its potential to cause disease, no effective treatments are available. This study investigates the vector competence of various mosquito species from temperate and tropical regions, to transmit BBKV. After mosquitoes were fed an infectious blood meal, various organs were collected at different time points to assess the infection status, viral replication, and infectiousness. The results showed that BBKV infection occurred in all tested mosquito species and persisted over time, with viral particles detected in mosquito or that can potentially be transmitted to vertebrate hosts via mosquito saliva. These findings highlight the broad vector competence of mosquitoes from diverse climatic regions for BBKV, indicating its potential risk for emergence and outbreaks in different regions of the world. The study emphasizes the need for enhanced surveillance and preventive measures to mitigate the potential threat posed by this emerging virus.

## Introduction

Alphaviruses are globally distributed, inhabiting all continents except Antarctica [1]. They are maintained in complex transmission cycles involving hematophagous arthropod vectors and a wide range of vertebrate hosts. Their host spectrum is remarkably broad, encompassing humans, non-human primates, horses, birds, amphibians, reptiles, rodents, pigs, marine mammals, and even salmonids. Based on their geographical distribution and associated clinical manifestations, alphaviruses are traditionally divided into Old World and New World groups [2]. New World alphaviruses, such as Venezuelan, Western, and Eastern equine encephalitis viruses, are primarily associated with encephalitic disease. In contrast, Old World alphaviruses,including Semliki Forest virus, Sindbis virus (SINV), Ross River virus (RRV), and Chikungunya virus (CHIKV),are generally associated with febrile and arthritogenic syndromes. Notably, SINV represents an exception to this dichotomy: although geographically restricted to the Old World, it phylogenetically clusters within the Western Equine Encephalitis (WEE) complex and can cause both encephalitic and arthritogenic disease [3,4].

Babanki virus (BBKV), a member of the WEE complex, is closely related to SINV [5]. Like other members of the *Togaviridae* family, BBKV is an enveloped virus with an icosahedral capsid and a positive-sense single-stranded RNA genome of approximately 11.7 kb encoding structural and non-structural proteins [6]. Multiple lines of evidence highlight its close evolutionary relationship with SINV. The BBKV Y-251 strain differs from the SINV prototype AR339 by only a limited number of amino acid substitutions and clusters within the African genotype I lineage of SINV, alongside isolates from South Africa and Uganda [7]. Consistently, recent sequence comparisons revealed very high nucleotide identity (up to 99.2%) between SINV isolates and BBKV strains [7,8]. Based on full genome analyses, BBKV has therefore been classified as a subtype of SINV within the WEE antigenic complex [9], underscoring their strong phylogenetic and evolutionary proximity.

Consistent with this genetic relatedness, BBKV also exhibits a broad ecological distribution. The virus was first isolated in 1969 from a pool of 55 adult *Mansonia africana* mosquitoes collected using human bait near *Raphia* palm trees in the Bambalang and Babanki regions of Cameroon [8], and subsequently amplified through intracerebral inoculation in newborn mice (Arbovirus Catalogue, Center for Disease Control - ArboCat, CDC). Since then, BBKV has been detected in multiple mosquito genera, including *Anopheles*, *Aedes*, *Culex*, *Eretmapodites*, *Coquillettidia*, and *Mansonia*, across sub-Saharan Africa and Madagascar [9–14]. In addition to entomological findings, BBKV has been isolated from humans in Senegal, Cameroon, Madagascar, and the Central African Republic [7,9], and neutralizing antibodies have recently been detected in bats in Uganda, further supporting its circulation in diverse vertebrate hosts [15]. Clinically, BBKV infection has been associated with febrile illness accompanied by rash and arthralgia [12]. Its detection across a wide geographic range, from West to East Africa, and its reported association with both mosquito and tick vectors (ArboCat, CDC), further highlight its potential for geographic expansion and emergence.

Despite this documented circulation across multiple vectors and hosts, the transmission dynamics and vector competence of BBKV remain poorly characterized. This represents a critical gap, particularly given its close phylogenetic relationship with SINV and its broad ecological distribution. Understanding its ability to infect, disseminate within, and be transmitted by mosquito vectors is essential for evaluating its emergence potential.

To address this, we assessed the vector competence of multiple *Aedes* and *Culex* species originating from both tropical and temperate regions, maintained under temperature conditions reflecting their natural environments. By comparing their ability to become infected, support viral dissemination, and transmit infectious particles, we aimed to determine whether BBKV can be efficiently transmitted across diverse ecological settings and to identify the most competent vector species within each climatic context.

## Methods

### Ethics statement

The blood meal feeding on living animals is approved by the ethics committee of Institut Pasteur C2EA n°89 under the agreement 2014-0068.

#### Cells.

VeroE6 cells (African green monkey epithelial cells) were maintained in culture with Dulbecco’s Modified Eagle Medium (DMEM) 1X + GlutaMAX (ref. 3166-021, Life Technologies) supplemented with 10% of heat-inactivated fetal bovine serum (FBS) (ref. A15-701, lot A70108-2369, PAA) and 1% of Penicillin-Streptomycin (P/S) (ref. 15140-122, Life Technologies) at 37°C with 5% CO_2_. C6/36 *Ae. albopictus* cells were cultured in Leibovitz’s L15 medium 1X (ref. 11415-049, Life Technologies) with 10% FBS and 1% P/S at 28°C without CO_2_.

#### Viral sequence.

The full length ARY168 11 724 bp DNA sequence was generated from a virus isolated from *Cx. decens* mosquito species (Yaoundé, Cameroon 1970] by Institut Pasteur of Dakar.

#### Mapping of ARY168 sequence.

Snapgene software was used to find open reading frames (ORF). Non-structural and structural proteins were determined based on the sequence alignment analyses with annotated reference sequences (BBKV HM147984.1 and SINV NC_001547.1) on Ugene.

#### Identification of predicted *E. coli* promoter sequences within BBKV genome ARY168.

The Neutral Network Promoter Prediction (NNPP) from the Berkeley *Drosophila* Genome Project (https://www.fruitfly.org/seq_tools/promoter.html) was used to identify predicted *E. coli* promotors (ECPs) sequences for cryptic expression of viral protein.

#### Add-ons.

A T7 promotor sequence was added at the 5’ UTR. A termination sequence including a hepatitis delta virus (HDV) antigenomic ribozyme and a simian virus (SV 40) polyadenylation signal (pA) (SV40pA) was placed at the 3’ UTR.

#### Fragmentation.

Mutated added-on BBKV sequence was divided into 4 fragments with a pace of approximately 3.000 nt framed by unique restriction sites.

#### Plasmid.

High copy number plasmid with ampicillin resistance gene pUC18 was chose for cloning. The initial multiple cloning site (MCS) of pUC18 was changed to ClaI-SpeI-PsiI-XhoI and NotI (32 nt) to a final pUC18_MSC_BBKV plasmid. To this aim, small oligos were hybridized at 95°C for 5 min and then cooled to room temperature. After purification, hybridized DNA was ligated to the open pUC18 plasmid with T4 ligase (ref. M0202, NEB).

#### Cloning.

The 4 synthetics genes were cloned into the pUC18_MSC_BBKV using both T4 ligase (ref. M020, NEB) or the recombinases In-Fusion HD Cloning Kit (ref. 638910, Takara)/Ezmax One-Step Cloning Kit (ref. 24303-2, Tolobio) strategies. Cloned plasmids were transformed into Top10 or JM110 competent bacteria. Mini cultures with DNA extraction were performed prior to maxi cultures and DNA extraction. Molecular clone was double checked by digestion restriction and by sequencing with BioSun company (China).

#### In vitro transcription.

Full length BBKV molecular clone DNA was linearized with NotI. After extraction with phenol-chloroform, Linear DNA was precipitated with ethanol and dissolved in RNAse free water. The *in vitro* transcription was achieved using mMESSAGE mMACHINE T7 (ref. AM1344, Invitrogen) kit following the manufacturer’s instructions. The transcript mRNA was precipitated by LiCl solution. RNA transcript was checked by electrophoresis and quantified.

#### Electroporation.

10 μg of full-length *in vitro* transcribed BBKV mRNA was electroporated into VeroE6 cells using the GenePulser XCell electroporation system (Bio-Rad) with 4 mm cuvettes and the following setting: 270V, 975 μF, ∞ Ω, 4 mm, 1 pulse. Cells were incubated at 37°C with 5% CO2 in a BSL-3 facility. After 4 hours post-electroporation, the cell's supernatant was removed and replaced with 2% FBS fresh cells media. The culture supernatant was collected 30h post electroporation when cytopathic effect (CPE) was observed, clarified by centrifugation, and re-amplified by infection of fresh VeroE6 or C6/36 cells. Cultured supernatant was harvested when CPE were present around 30 hours post amplification), clarified by centrifugation, aliquoted and stored at -80°C.

#### Titration by plaque assay.

The produced virus was 10-fold diluted until 10^-10^. VeroE6 cells at 90% confluence were infected with 200 μL of virus dilutions in DMEM supplemented with 2% of FBS in the BSL-3 environment. After 1 hour, the inoculum was removed and replaced with 2% final concentration carboxymethyl cellulose (CMC) with DMEM and 2% FBS 1% P/S media. After 24 hours at 37°C with 5% CO2, cells were washed from the CMC polymer mix with PBS and fixed with a 4% PFA solution during 30 min. After washing the PFA with PBS, the plate was stained with crystal violet 0.1% solution. Plaque per dilution were counted to compute the viral titer that is expressed as plaque forming unit per ml (pfu/ml). Viral stocks were generated from the molecular clone in both insect and mammalian cells. The stock produced in insect cells was selected for the experiments, based on previous findings showing that SINV produced in insect cells is more infectious for mosquitoes [16].

#### Antibodies.

Noncommercial mouse polyclonal antibodies against SINV were used as primary antibodies. Mouse polyclonal antibodies against *Ae. aegypti*, *Ae. albopictus* and *Cx. pipiens* salivary glands proteins were used as primary antibodies.

Calnexin polyclonal antibody purified from rabbit serum (ADI-SPA-865, Enzo Life Sciences) was used as a primary antibody to detect housekeeping genes in mammals.

Both Goat anti-Mouse IgG (H + L) HRP conjugated (Cat#172-6516 Bio-Rad) and Goat anti- Rabbit (Cat#172-6515 Bio-Rad) were used as a secondary antibody for chemiluminescence. For fluorescent microscopy, Alexa Fluor 488 AffiniPure Goat anti-mousse IgG (H + L) Jackson ImmunoResearch (ref. 115-545-003) was used.

#### Mosquitoes strain.

*Aedes aegypti* (PAEA) strain is a laboratory colony (more than 400 lab- generations) isolated in 1960 in French Polynesia. *Aedes albopictus* Nice strain was collected in Nice, South of France in 2011. *Aedes albopictus* Saint-Benoît strain was collected in Saint-Benoît, Réunion Island in the Pacific Ocean in 2012. *Culex pipiens molestus* Paris strain was collected in Paris in 2017. For the remainder of the manuscript, this population will be referred to as *Cx. pipiens* Paris. *Culex quinquefasciatus* (Slab strain) was kindly donated by Mylène Weill from the Institute of Evolution Sciences of Montpellier (ISEM) (University of Montpellier, CNRS, IRD, EPHE, CIRAD, INRAP).

#### Mosquito breeding.

From the egg to the female adult for blood meal, it takes around 4 weeks. Every stage of the mosquito development takes place in a room at 27°C, 70% of relative humidity with a 12-hour photoperiodicity. *Aedes* eggs are stored on dry blotting forest green paper up to 3 months whereas *Culex* eggs cannot be stored. Mosquito eggs are put in 2 L of non-limestone water with cats and fish dry food. Depending on the species, dry brewer’s yeast can be added to boost the development. Larvae from stage 1 to 3 are reared in the same condition in netted- covered flat bucket. Pupae are sorted and placed in a netted cage before emergence. Adult male emergence happens few days before the female one. Adult mosquitoes are fed *ad libitum* on 10% sucrose solution. Two blood meals at 3 days apart are necessary for the females to lay eggs on water (*Culex*) or blotting paper (*Aedes*). For this purpose, mice are anesthetized with an injectable solution of ketamine/xylazine mix and ventral sided placed on the top of the netted cage for 20 min.

#### Exposure to infectious blood meals.

Week-old female mosquitoes were sorted and starved 24 hours prior to the infectious blood meal. On the infection’s day, fresh rabbit blood was collected on pre-heparinized 15 mL tubes and washed 3 times with PBS. The infectious blood meal was constituted in the BSL-3 facility and consists in one third of viral suspension (final concentration of 1.10^8^ PFU/mL), two thirds of rabbit erythrocytes and adenosine triphosphate (ATP) as a phagostimulant to a final concentration of 10 mM. *Aedes* species were allowed to feed for 20 minutes through a collagen membrane (*Ae. aegypti*) or swine intestine (*Ae. albopictus*) covering electric feeders maintained at 37°C (Hemotek system). *Culex* species were allowed to feed for 30 minutes directly on blood-soaked cotton at 37°C in an incubator with 5% CO_2_ and without light. After feeding, mosquitoes are anesthetized at 4°C. Blood-fed female mosquitoes are selected on ice and transferred into netted-cardboard boxes with *ad libitum* 10% sucrose solution and kept in an incubator. After infection, incubation parameters were chosen according to environmental condition of each mosquito species in nature with 80% of humidity and a 12-hour photoperiodicity incubator for a maximum duration of 14-days. Temperature of 28°C was used for tropical mosquito species such as *Cx. quinquefasciatus, Ae. aegypti* and *Ae. albopictus* St-Benoit. Other mosquito species such as *Cx. pipiens molestus* Paris strain*, Ae. albopictus* Nice are considered temperate and were incubated at 24°C.

#### Mosquito sample dissections.

In the BSL-3 facility, mosquitoes were anesthetized on ice at 7- and 14 days after the infectious blood meal exposition. They went through a 70% ethanol bath and a PBS one. They were dissected in a drop of PBS under a magnifying glass with micro dissecting needles and micro dissecting needle holders (Fine Science Tools, Foster City, USA) and tweezers (#11252-23, Dumont tweezer - Style 5 - Dumoxel). For RNA extraction, midguts, legs and salivary glands were collected from the same mosquito and separately homogenized in 350 μL of RA1 lysis buffer from Nucleospin RNA kit (ref. 740955.50 Macherey-Nagel) with 1% of 𝛽-Mercaptoethanol (M6250, Sigma-Aldrich) and sterile glass beads (ref. 152018, Dutscher) and then stored at -80°C. For infectious particles quantification, midguts and salivary glands were collected from the same mosquito and separately homogenized in 500 μL of DMEM 2% FBS 1% P/S and 1X Antibiotics (ATB)/Antifungal (ATF). Homogenization was performed by the Precellys system (Bertin Technologies) at 10 000 *x g* 15 sec twice with a 10 sec break and kept at -80°C. For viral proteins detection, midguts and salivary glands from the same mosquito were collected and individually stored in 10 μL of RIPA 2X buffer (ref. RB4476, BioBasic Canada) at -20°C.

#### Saliva sample collection.

Legs from mosquitoes were first removed. Then, the mosquito proboscis was inserted in 10 μL tips pre-filled with PBS. From this saliva collection per mosquito, the sample is split in three. Saliva samples for RNA extraction were lysed in 100 μL of RA1 lysis buffer and 2 μL of reducing agent TCEP (Tris(2-carboxyethyl) phosphine) from Nucleospin RNA XS kit (ref. 740902.50 Macherey-Nagel). Samples for viral protein detection were mixed with a 1:1 ratio of RIPA 2X buffer (ref. RB4476, BioBasic Canada) and kept at -20°C. The remaining volume was used with DMEM 2% FBS 1% P/S and 1X ATB/ATF for quantification of infectious particles.

#### Viral RNA quantification by RT-qPCR analysis.

*RNA extraction.* After lysis, total RNA was extracted using the Nucleospin RNA kit (ref. 740955.50 – Macherey-Nagel) following manufacturer’s instructions. *Reverse transcriptase (RT) and quantitative PCR (qPCR).* Viral RNA quantification was performed using 2 μL of extract RNA with the *Power* SYBR Green RNA-to-CTTM *1- Step* Kit (ref. 4389986, ThermoFisher Scientific) following the manufacturer’s instructions. Specific primers were used to amplify the viral RNA nsp2-Forward: AAGCGATGCGTTAAGAAGGA and nsp2-Reverse: ACTTGATGATAGCTGACTTGCC.

The QuantStudio 6 Flex Real-Time PCR System (Applied Biosystem) monitors the fluorescent signal produced by the SYBR Green which binds double-stranded DNA. The thermal cycling condition are: reverse transcription for 30 min at 48°C; 10 min at 95°C and then 40 cycles of 15 sec at 95°C followed by 1 min at 60°C. Quantity of RNA copies per mL of sample was extrapolated from the standard curve generated from the 10-fold serial dilution of the *in vitro* transcript mRNA previously quantified by the ND-1000 Spectrophotometer (Thermos Fisher Scientific).

#### Viral protein detection by Western Blot.

After thawing, midguts and salivary glands samples collected in RIPA buffer were centrifuged at maximum speed during 5min at 4°C. Then samples were denatured in 1X NuPage LDS Buffer (ref. NP0008, ThermoFisher Scientific) during 5 min at 95°C and loaded to a precast Nu-Page 4–12% Bis-Tris Gel (ref. NP0322BOX, ThermoFisher Scientific). Proteins were separated by electrophoresis at 60 mA and transferred thanks to a Trans-Blot Turbo Mini PVDF Transfer Packs (ref. 1704156, Bio-Rad) with the Trans-Blot Turbo System (Bio-Rad).

#### Viral protein detection by Dot blot.

An Immuno-blot PVDF membrane (ref. 1620177; Bio-Rad) was activated by submerging it in 100% ethanol and rinse it with PBS. Then, we applied 2 μL of each saliva sample in the RIPA buffer in one square. The blot was then air-dried for 5–10 min. After blocking with PBS – Tween 20 0.1% (ref. P7949, Sigma Aldrich) - Skim Milk 5% (ref. 232100, BD) during 1 hour at room temperature under agitation, the PVDF membrane was incubated overnight at 4°C with primary antibody diluted in blocking buffer under agitation. Finally, the membrane was incubated with the secondary antibody diluted in the blocking buffer during 1 hour at RT under agitation. Proteins were revealed using Pierce ECL Western Blotting Substrate (ref. 32106, ThermoFisher Scientific) and MyECL Imager (ThermoFisher Scientific).

#### Results interpretation.

To determine vector competence of each mosquito species, infection, dissemination and transmission rates are computed according to the titration by plaque assay results (from the midguts and salivary glands in DMEM buffer) and the following formulas [17]:


$$\begin{array}{c} Feeding\;rate\;=\frac{mosquitoes\;fed}{total\;mosquitoes\;exposed\;to\;the\;infectious\;blood\;meal} \end{array}$$

$$\begin{array}{c} Infectious\;rate=\frac{positive\;midguts}{total\;mosquitoes\;fed} \end{array}$$

$$\begin{array}{c} Disse\text{min}ation\;rate\;=\frac{positive\;legs\;or\;salivary\;glands}{positive\;midguts} \end{array}$$

$$\begin{array}{c} Transmission\;rate\;=\frac{positive\;saliva}{positive\;salivary\;glands} \end{array}$$

### Biostatistical analysis

Differences in infection and engorgement rates among mosquito species were initially evaluated using a Chi-square test. When the overall test was significant (p < 0.05), pairwise Chi-square tests for comparisons between species were conducted. P-values were adjusted for multiple comparisons using the Bonferroni correction.

Statistical analyses on RT-qPCR and titration results were computed using Simstat software (v.2.5.8). Nonparametric Krustal-Wallis and Mann Whitney tests were used to test differences between groups. P-values were adjusted for multiple comparisons when appropriate (Tukey’s range test).

Mosquito species were maintained under temperature conditions reflecting their natural ecological environments. Therefore, comparisons between species are intended to reflect their relative performance within their ecological niches rather than direct comparisons under standardized laboratory conditions.

## Results

### Infectious molecular clone construction

To reduce experimental variability and enable controlled comparisons, we generated a molecular clone of BBKV. Molecular clones have proven highly valuable in alphavirus research, as demonstrated for virus and CHIKV [18,19]. The BBKV clone developed here provides a robust and reproducible tool for infection studies and establishes a foundation for future molecular systems—such as replicons, pseudoparticles, or virus-like particles—to investigate viral tropism and other biological properties.

### Identification of predictive *E. coli* promoter sequences within BBKV genome ARY168

The full-length genome BBKV ARY168 sequence was kindly provided by IP Dakar. Non-structural and structural proteins were determined on the 2 open reading frames (ORF) based on the sequence alignment analyses with annotated reference sequences (BBKV HM147984.1 and SINV NC_001547.1) on Ugene. Based on the previous work done for Yellow fever and Japanese encephalitis viruses by the team of Pu *et al.* [20], putative predicted ECPs sequences for cryptic expression of viral protein were found using the NNPP from the Berkeley *Drosophila* Genome Project (https://www.fruitfly.org/seq_tools/promoter.html). In total, 14 putative predicted ECPs with a score higher than 0.9 were identified for the BBKV genome ARY168 sequence represented in Table 1.

**Table 1:** Predicted *E. coli* promoters (ECPs) found for ARY168.

Start	End	Score	Promoter Sequence
27	72	0.94	TATTGAATCAAACAGCCGACCAATTGCACTACCATCACAATGGAGAAGCC
252	297	1.00	ATTTTGGACATAGGCAGCGCACCGGCTCGTAGAATGTTTTCCGAGCACCA
1016	1061	0.93	AGTTACTGACACAGTAAAAGGAGAACGGGTATCGTTCCCTGTGTGCACGT
3295	3340	0.97	TGTTTTCCAAACAGAGCATCCCGCTAACGTACCATCCCGCCGATTCAGCG
4056	4101	0.93	AATTGCGTGATTTCGTCCGTGTACGAGGGTACAAGAGACGGAGTAGGAGC
4653	4698	0.91	AGTTGCTTGAAGGGAAGAAAGGGATTCAGTACTACAAAAGGAAAGTTGTA
5280	5325	0.90	AGTTGGTCGTCAGGACCTAGTTCGCTAGAGATAGTAGACCGAAGGCAGGT
5655	5700	0.95	TCATTTGAACCGGGCGAAGTGAACTCAATTATATCGTCCCGATCAGCCGT
6226	6271	0.96	ATATGGTAGACGGGACAGTCGCTTGCCTGGATACTGCAACCTTTTGCCCC
7166	7211	0.96	TATTGGCGACGACAACATCATACACGGAGTAGTATCTGACAAAGAAATGG
7985	8030	0.94	AGTTGGAGGCCGACAGATTGTTCGACGTCAAAAATGAGGATGGAGACGTC
8091	8136	0.92	AACTATTGACCACCCTGTGCTATCAAAGCTCAAATTCACCAAGTCGTCAG
8675	8720	0.94	CATATTTGGGCACATGCTCGTACTGTCACCATACTGAACCGTGCTTTAGC
10128	10173	0.98	ACTTGTTGAAAGGGCAGGGTACGCCCCGCTCAATTTGGAGATCACTGTCA

Transcription start is shown in larger font.

Predicted ECPs found for ARY168 using the NNPP from the Berkeley Drosophila Genome Project (https://www.fruitfly.org/seq_tools/promoter.html).

### BBKV molecular clone construction *in silico*

**Fig 1:**
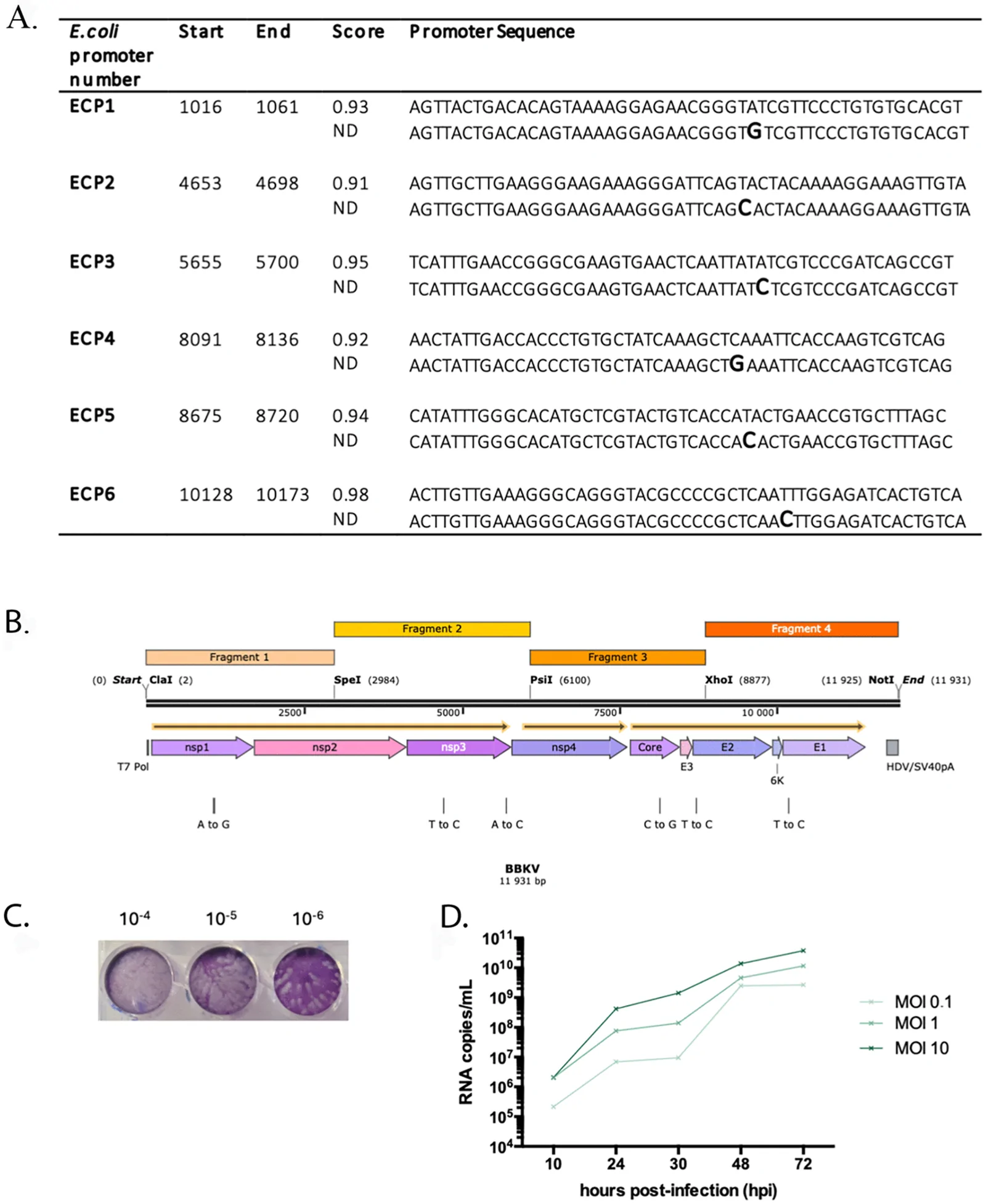
BBKV molecular clone construction and validation. **A.** Silent mutations introduced in ECPs in bold letters. New scores were not detectable (ND) with the NNPP. **B**. Annotated map of mutated BBKV ARY 168 genome with Snapgene and Ugene softwares. **C**. Observation of Plaque Forming Unit on infected VeroE6 cells at different BBKV dilutions. **D**. Replication kinetics of BBKV on C6/36 cells at a multiplicity of infection (MOI) of 0.1; 1 and 10. Total RNA was quantified at different time points in cell supernatant.

Silent mutation introduction in the BBKV genome did not affect the amino acid sequence of ARY168 genome. Silent mutations were manually introduced to lower the score to 0, except in the 5’ and 3’ UTR region and the non-coding part between the structural and non-structural protein genes. In total, 6 ECPs scores were lower to non-detectable (ND) keeping the same amino acid sequence (Fig 1A). These mutations were generated to improve the stability of the BBKV genome in bacteria when amplified.

Using SnapGene in silico, a specific T7 promotor sequence was added at the 5’UTR and a termination sequence including a hepatitis delta virus (HDV) antigenomic ribozyme [21] and a simian virus (SV 40) polyadenylation signal (pA) (SV40pA) [22] was placed at the 3’ UTR. Combination of HDV ribozyme and SV40pA cassette would allow to generate more efficient gene expression [23].

Unique restriction sites were introduced for the cloning of the mutated full-length BBKV genome that was fragmented into 4 sections of 3.000 nt approximately (Fig 1B).

### BBKV molecular clone construction and validation

High copy number pUC18 plasmid contains an ampicillin resistance gene that will be used as a selection gene by ampicillin antibiotic. pUC18 original multiple cloning site (MSC) was replaced by MSC_BBKV (ClaI-SpeI-PsiI-XhoI-NotI) allowing the cloning of BBKV synthetic gene fragments manufactured by Generay Company (China). The final full-length construction was confirmed by digestion restriction and by sequencing analysis. Plaque forming unit from the viral stock on VeroE6 cells (Fig 1C). Replication kinetics was also tested on C6/36 cells (Fig 1D).

### Vector competence

#### Mosquito feeding rate.

Mosquitoes were exposed to an infectious BBKV blood meal at a viral titer of 1x10^8^ PFU/mL. The viral titer was double checked after the meal exposition by titration assay. The feeding rate was calculated as the proportion of mosquitoes that ingested the blood meal over the number of total mosquitoes exposed to the infectious blood meal (Table 2). Significant variation was observed among mosquito species (p < 0.00001), with *Cx. quinquefasciatus* exhibiting the highest feeding rate (95%), while *Ae. aegypti* had the lowest (48%). *Aedes albopictus* (Nice and Saint-Benoît) showed similar feeding rates (~60%), whereas *Cx. pipiens* had a moderate feeding rate (82%) (S1 Table).

**Table 2:** Feeding rate after the exposition to BBKV infectious blood meal.

Mosquito species	Feeding method	Feeding rate (%)
*Culex pipiens* Paris	On cotton	382/465 (82%)
*Aedes albopictus* Nice	Hemotek	327/550 (59%)
*Culex quinquefasciatus*	On cotton	425/447 (95%)
*Aedes albopictus* Saint-Benoît	On cotton	376/650 (58%)
*Aedes aegypti* PAEA	Hemotek	288/600 (48%)

The feeding rate represents the number of fed mosquitoes over the number of total mosquitoes exposed to the infectious blood meal at 1.10^8^ PFU/mL.

### Infection and persistence

**Fig 2:**
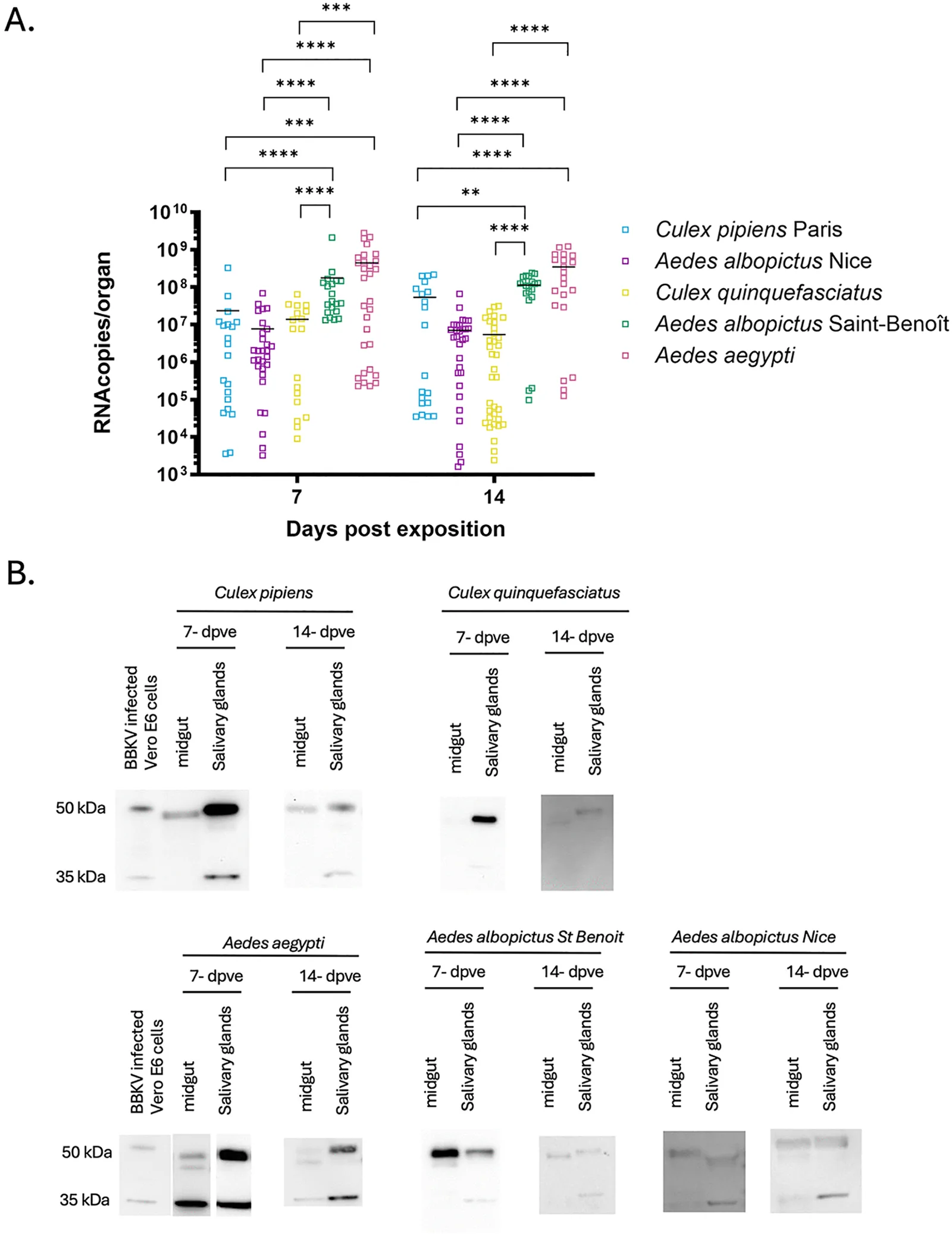
Viral amplification and detection at 7- and 14-dpve. **(A)** Viral RNA amplification for genome quantification by RT-qPCR in mosquito midguts for BBKV. At 7-dpve, n = 30 for *Ae. albopictus* Nice and *Ae. aegypti*; n = 20 for *Cx. quinquefasciatus*, *Ae. albopictus* Saint-Benoît and *Cx. pipiens* Paris. At 14-dpve, n = 20 for *Cx. pipiens* Paris and *Ae. albopictus* Saint-Benoît; n = 22 for *Ae. aegypti*; n = 34 for *Ae. albopictus* Nice and n = 40 for *Cx. quinquefasciatus*. **: p < 0.01; ***: p < 0.001; ****: p < 0.0001 (Nonparamatric Krustal-Wallis and Mann-Whitney tests, Tukey p-value correction). **(B)** BBKV viral protein detection in the MG and SG. Mosquito organs were collected in the RIPA buffer. After SDS-PAGE electrophoresis and transfer to a PVDF membrane, SINV ascites were used. Bands at 35 kDa represent core protein and 50 kDa preE1-E2.

At 7 days post–viral exposure (dpve), viral RNA was detected in the midguts of all mosquito groups (Fig 2A). Overall, viral loads were heterogeneous across species. *Ae. aegypti* and *Ae. albopictus* Saint-Benoît mosquitoes showed the highest mean RNA levels (>108 copies/midgut). In contrast, *Culex* and *Ae. albopictus* mosquitoes displayed significantly lower midgut RNA copy numbers (p < 0.0001). Within *Ae. aegypti* (n = 30), three distinct midgut viral load categories were observed: high, intermediate, and low. For *Ae. albopictus* Saint-Benoît, all individuals belonged to a single high-load group, with viral RNA levels exceeding 10⁷ copies per midgut.

At 14-dpve, midgut viral loads differed significantly across all mosquito species (p < 0.0001), with the highest levels observed in *Ae. aegypti* and *Ae. albopictus* (Saint-Benoît), and the lowest in *Cx. quinquefasciatus* and *Ae. albopictus* (Nice) (S2, S3, S4, S5, S6, S7 Tables).

At 14-dpve, *Cx. pipiens* Paris, *Cx. quinquefasciatus*, *Ae. albopictus* Saint-Benoît and *Ae. aegypti* had a heterogeneous distribution with a high pool around 10^7^-10^8^ RNA copies/midguts and a lower one at 10^4^-10^5^ RNA copies/midgut. However, *Ae. albopictus* Nice midgut viral RNA had a homogeneous distribution ranging from 10^3^ to 10^8^ RNA copies/midguts.

Viral loads at 7- or 14-dpve were non-significantly different within the same species. If we compared the viral loads of the same climate area species, *Cx. pipiens* from Paris and *Ae. albopictus* from Nice were not significantly different) (S1, S2, S3, S4, S5, S6, S7 Tables).

Western blot analysis of mosquito midguts using anti-Sindbis virus antibodies revealed the presence of viral structural proteins, including the envelope glycoproteins preE1–E2 (~50 kDa) and the capsid protein (~35 kDa) at both 7- and 14-dpve (Fig 2B). Detection patterns varied between mosquito species. A double band around ~50 kDa was observed in several *Aedes* midgut samples, whereas a single band at a similar molecular weight was detected in some *Culex* samples. The band corresponding to the capsid protein (~35 kDa) was inconsistently detected across mosquito species (Fig 2B) (S1, S2, S3, S4, S5, S6 Figs, S8 Table).

### BBKV replication in the mosquito midgut and persistence

**Fig 3:**
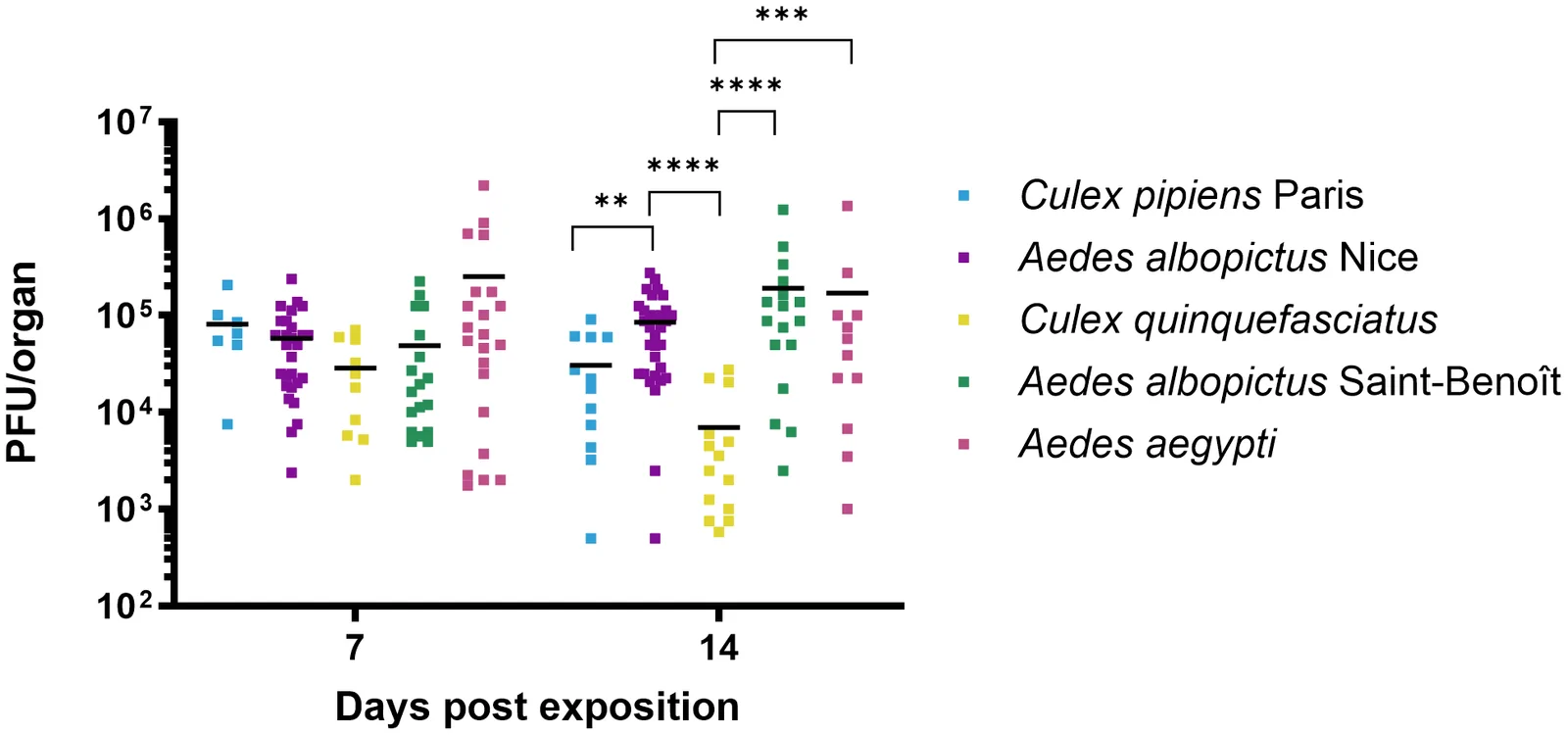
Viral titer in MG at 7- and 14-dpve. After the infected artificial blood meal, MG from the different mosquito species were collected and viral replicative particles were quantified by plaque assay titration on VeroE6 cells (n = 40 for all mosquito species). *: p < 0.05; **: p < 0.01; ***: p < 0.001; ****: p < 0.0001 *(*Krustal-Wallis and Mann-Whitney tests, Tukey p-value correction)*.*

Infectious particles or replicating viruses were quantified in the midguts of mosquitoes at 7- and 14-dpve (Fig 3). No significant difference was found between all mosquito species at 7-dpve whereas a high significant difference was found between viral titers measured in the midguts of the different species at 14-dpve. The viral titer found in *Ae. albopictus* Nice, *Ae. albopictus* Saint-Benoît and *Ae. aegypti* midguts were similar and significantly higher than those of *Cx. pipiens* and *Cx. quinquefasciatus*. We then compared the difference of viral replication between 7-and 14-dpve. A significantly decreasing viral titer was found in *Cx. pipiens* and *Cx. quinquefasciatus* midguts between the two time points (p < 0.04 and p < 0.0065 respectively). Interestingly, a significant increase of the viral titer was observed for *Ae. albopictus* Saint-Benoît (p < 0.024) whereas similar titers are observed for *Ae. albopictus* Nice and *Ae. aegypti* (S9 Table).

The infection rates at 7- and 14-dpve (Table 3) were significantly different according to the different mosquito species (p = 0.00003 and p = 0.00006 respectively). Regarding the results at 7-dpve, *Cx. pipiens* mosquitoes had a lower rate of infection than both populations of *Ae. albopictus* (p = 0.0004 for *Ae. albopictus* Nice and for *Ae. albopictus* Saint- Benoît). The rate of infection of the three *Aedes* mosquitoes was not significantly different. The rate of infection of *Cx. quinquefasciatus* was similar to that of *Cx. pipiens*. In conclusion, the *Aedes* mosquitoes had a higher infection rate than the *Culex* mosquitoes tested.

**Table 3:** Infection rates at 7- and 14- dpve.

Mosquito species	Infection rate % 7- dpve	Infection rate % 14-dpve
*Culex pipiens* Paris	39% (7/18)	60% (12/20)
*Aedes albopictus* Nice	93% (28/30)	82% (33/40)
*Culex quinquefasciatus*	50% (10/20)	35% (14/40)
*Aedes albopictus* Saint-Benoît	95% (17/18)	85% (17/20)
*Aedes aegypti* (PAEA)	73% (22/30)	55% (12/22)

At 14-dpve, the infection rates of *Ae. albopictus* strains, *Ae. aegypti* and *Cx. pipiens* were similar. The rate of infection of *Cx. quinquefasciatus* mosquitoes dropped and was significantly inferior to that of the two populations of *Ae. albopictus* (p = 0.00002 for the European strain and p = 0.0003 for the tropical strain).

The infection rate represents the number of infected mosquitoes over the number of fed mosquitoes exposed to the infectious blood meal at 7- and 14-dpve.

### BBKV dissemination to the mosquito leg and persistence

**Fig 4:**
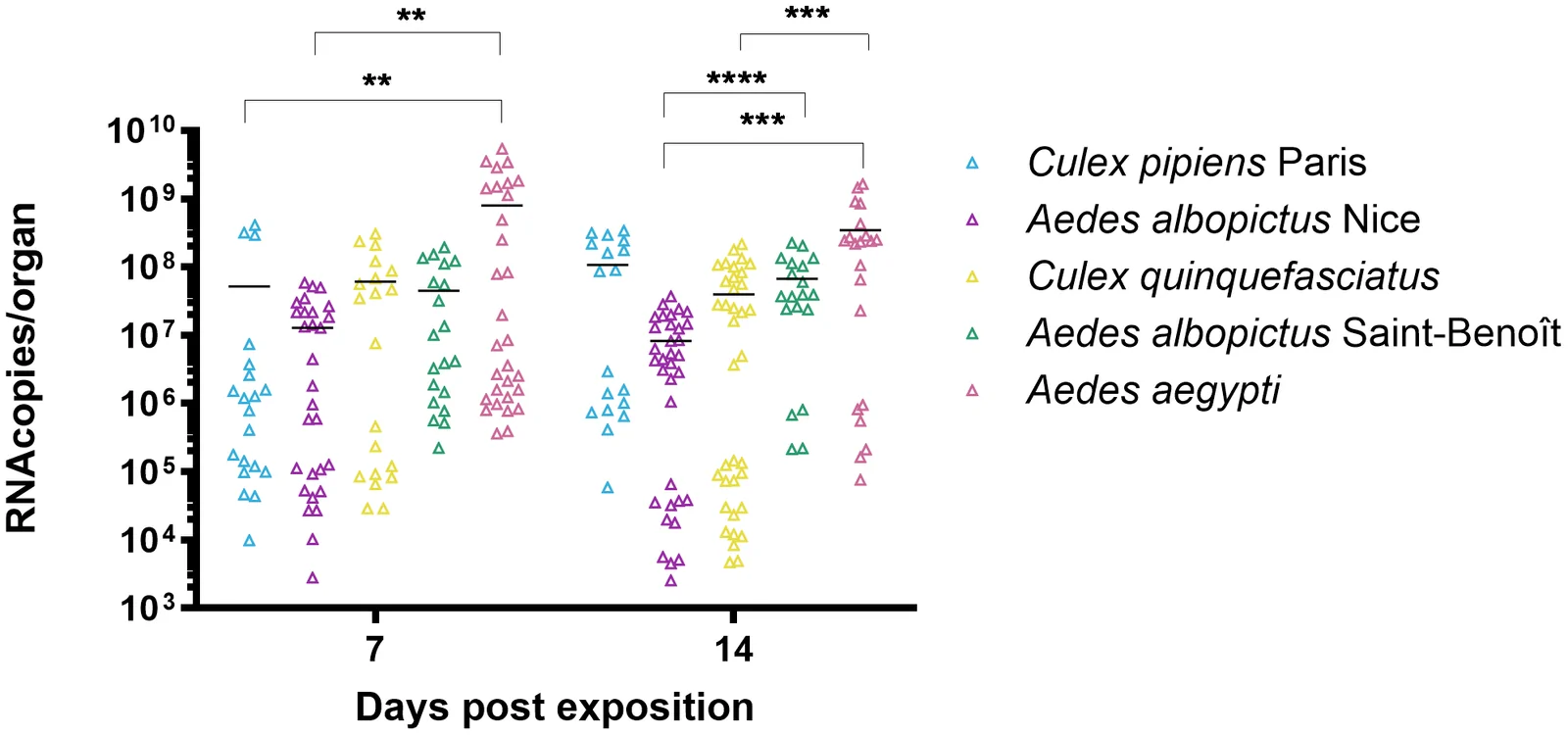
Viral RNA amplification for genome quantification by RT-qPCR in the legs at 7- and 14-dpve. At 7-dpve, n = 30 for *Ae. albopictus* Nice and *Ae. aegypti*; n = 20 for *Cx. quinquefasciatus*, *Ae. albopictus* Saint-Benoît and *Cx. pipiens* Paris. At 14-dpve, n = 20 for *Cx. pipiens* Paris and *Ae. albopictus* Saint-Benoît; n = 22 for *Ae. aegypti*; n = 34 for *Ae. albopictus* Nice and n = 40 for *Cx. quinquefasciatus*. *: p < 0.05; **: p < 0.01; **: p < 0.001; ****: p < 0.0001 (Krustal-Wallis and Mann-Whitney tests, Tukey p-value correction)*.*

Viral RNA was detected in mosquito legs at 7- and 14-dpve to confirm the BBKV dissemination from the midgut escape barrier and persistence. There was a heterogeneous distribution of the viral RNA in all the species studied: a very high pool and a lower one (Fig 4). As for the midguts, there was no significant difference within each species between 7- and 14-dpve. RT-qPCR Analysis of viral RNA in mosquito legs at 7-dpve reveals higher levels in *Ae. aegypti* compared to *Ae. albopictus* (Nice) and *Cx. pipiens* (p < 0.005). For *Cx. quinquefasciatus* and *Ae. albopictus* from Nice, the viral load in the legs was significantly lower compared to *Ae. aegypti* (p < 0.001) at 14-dpve. There was also a significant difference (p < 0.05) between *Ae. albopictus* from Réunion Island and *Ae. albopictus* Nice at 14-dpve.

### BBKV dissemination to the mosquito salivary glands and persistence

**Fig 5:**
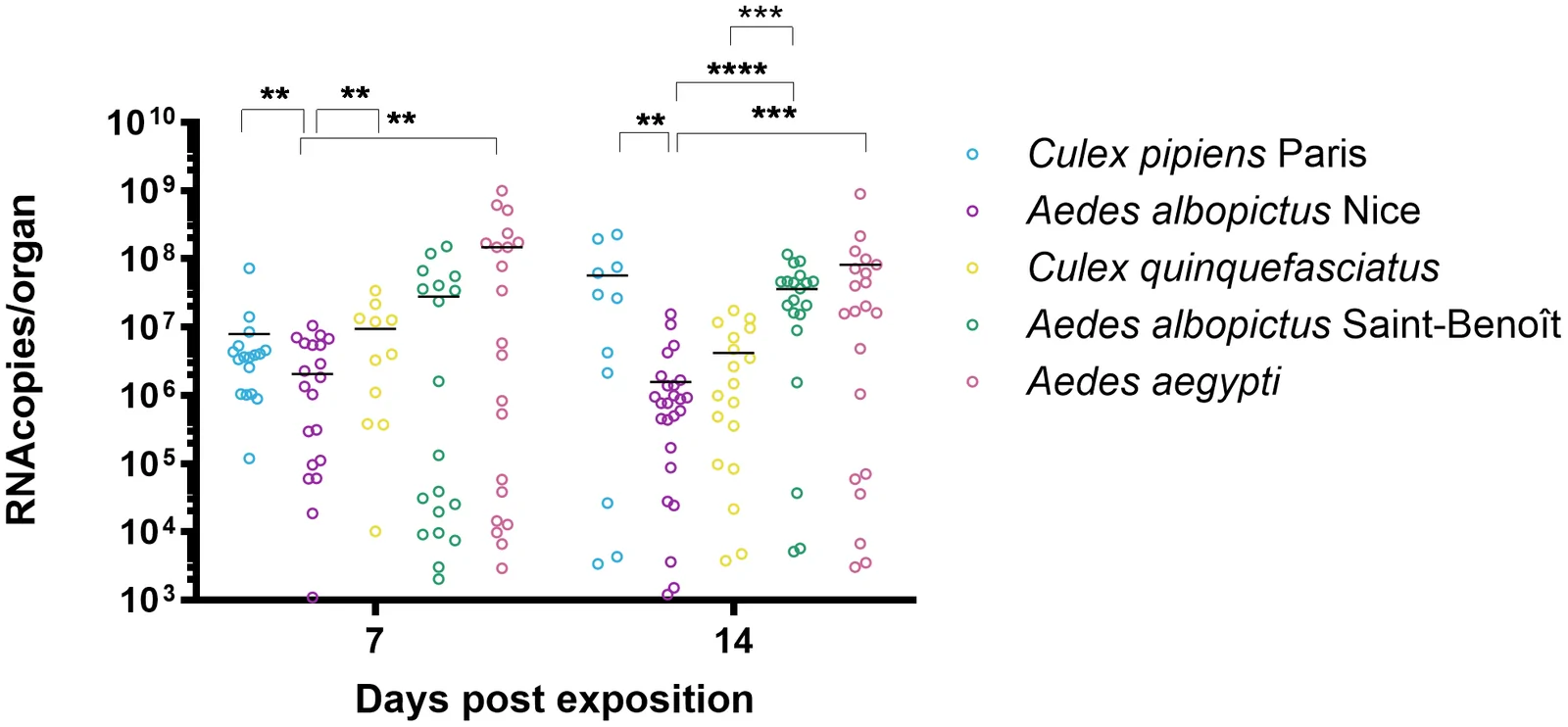
Viral RNA amplification for genome quantification by RT-qPCR in the salivary glands at 7- and 14-dpve. At 7- dpve, n = 30 for *Ae. albopictus* Nice and *Ae. aegypti;* n = 20 for *Cx. quinquefasciatus, Ae. albopictus* Saint-Benoît and *Cx. pipiens* Paris. At 14-dpve, n = 20 for *Cx. pipiens* Paris and *Ae. albopictus* Saint-Benoît; n = 22 for *Ae. aegypti;* n = 34 for *Ae. albopictus* Nice and n = 40 for *Cx. quinquefasciatus.* ns or not represented: non significative; *: p < 0.05; **: p < 0.01; ***: p < 0.001; ****: p < 0.0001 (Kruskal-Wallis and Mann-Whitney tests, Tukey p-value correction).

Viral RNA was extracted from salivary glands collected on the same mosquitoes for which we previously analyzed midguts and legs at both 7- and 14-dpve and analyzed by RT-qPCR (Fig 5). BBKV viral RNA genomes were detectable at both 7- and 14-dpve in salivary glands of mosquito species.

Viral RNA genome levels were not significantly different in the SG at 7- and 14-dpve within each species. At 7-dpve, viral RNA copy levels in the SG of *Ae. albopictus* Nice was significatively inferior to that of *Cx. pipiens* Paris, *Ae. aegypti and Cx. quiquefasciatus* (p < 0.005). At 14-dpve, the amount of RNA copies per pair of salivary glands was significantly higher (p < 0.005) for *Cx. pipiens* Paris, *Ae. aegypti* and *Ae. albopictus* Réunion Island than *Ae. albopictus* Nice (p < 0.005). The amount of viral RNA copies was also significantly (p < 0.005) higher in *Ae. albopictus* Saint-Benoît than in the *Cx. quinquefasciatus* population) (S2, S3, S4, S5, S6, S7 Tables).

Western blot analysis of mosquito salivary glands using anti-Sindbis virus antibodies revealed the presence of viral structural proteins at 7- and 14-dpve (Fig 1B). A band at ~35 kDa, corresponding to the capsid protein, was detected in several samples. In addition, in some salivary gland samples, a band or a double band around ~50 kDa was observed, consistent with the preE1–E2 envelope glycoproteins. Detection patterns varied across mosquito species and individual samples (S1, S2, S3, S4, S5, S6 Figs, S8 Table).

### Replication in the salivary glands

**Fig 6:**
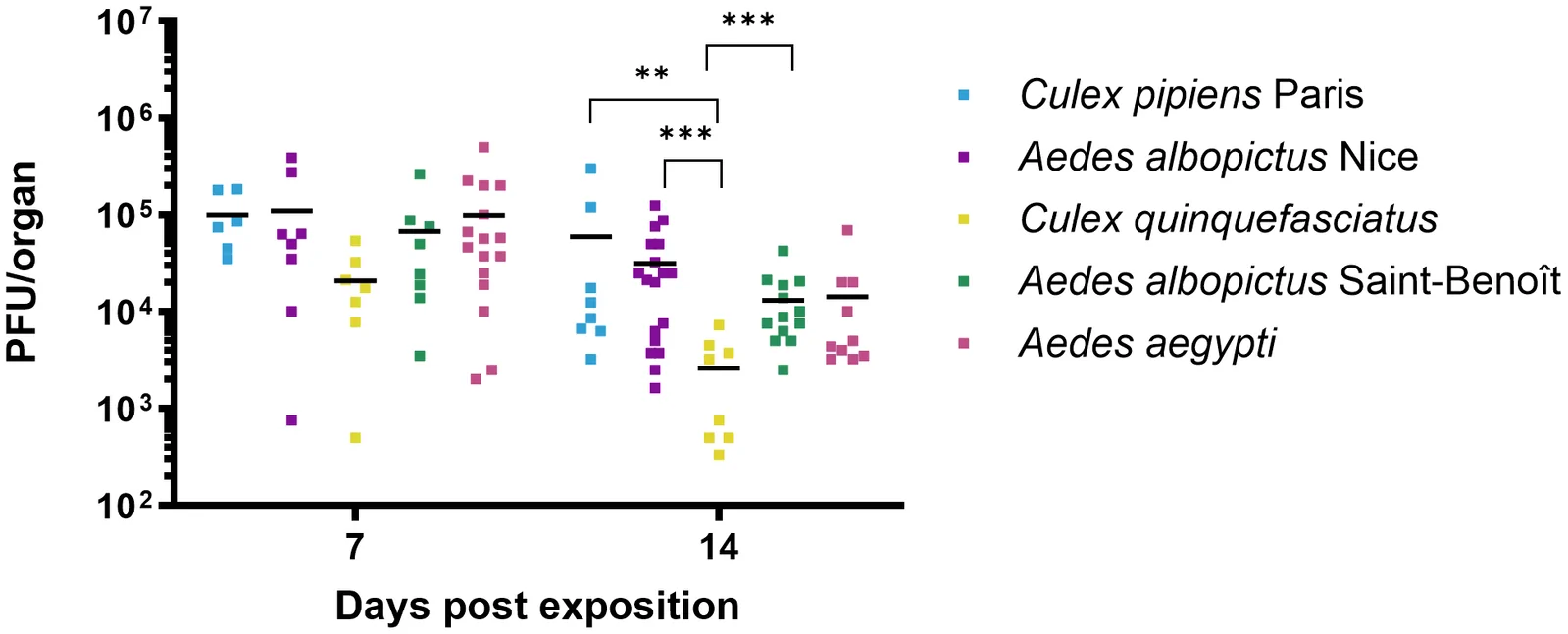
Viral replicative particles quantification in salivary glands. **Salivary glands were collected at 7- and 14-dpve.** Viral replicative particles were quantified by titration plaque assay on VeroE6 cells (n = 40 for all mosquito species). ns or not represented: non significative; *: p < 0.05; **: p < 0.01; ***: p < 0.001; ****: p < 0.0001 (Kruskal-Wallis and Mann-Whitney tests, Tukey p-value correction).

Salivary glands were collected (from the same mosquito that were previously used for viral titer analysis) at 7-and 14-dpve and the viral titer was analyzed (Fig 6). No significant difference was observed at 7-dpve between the different species.

Infectious particles measured in *Cx. pipiens*, *Ae. albopictus* Nice, *Ae. albopictus* Saint-Benoît salivary glands were similar and significantly higher than those of *Cx. quinquefasciatus* at 14-dpve (p < 0.005).

A significant difference in the salivary glands titers was observed between 7- and 14- dpve for *Cx. quinquefasciatus, Ae. albopictus* Saint-Benoît *and Ae. aegypti* (p < 0.01; p < 0.05; p < 0.01) for which the viral titers decreased at 14-dpve compared to 7-dpve (S9 Table).

Using this dataset, the dissemination rate to the salivary glands can be determined by the ratio of infected salivary glands to infected midguts (Table 4). At 7-dpve, a significant difference was observed between all species (p < 0.0003), driven by the low dissemination rate in *Ae. albopictus Nice*. No significant differences were found among the other species. At 14-dpve, no significant differences were observed. However, for *Ae. albopictus Nice*, the dissemination rate was higher at 14-dpve than at 7-dpve (p < 0.008), consistent with the increased viral replication in the midgut at this time point.

**Table 4:** Dissemination rate to the salivary glands.

Mosquito species	Dissemination rate % 7-dpve	Dissemination rate % 14-dpve
*Culex pipiens* Paris	86% (6/7)	67% (8/12)
*Aedes albopictus* Nice	21% (6/28)	55% (18/33)
*Culex quinquefasciatus*	70% (7/10)	57% (8/14)
*Aedes albopictus* Saint-Benoît	59% (10/17)	76% (13/17)
*Aedes aegypti* (PAEA)	77% (17/22)	83% (10/12)

The dissemination rate represents the number of infected salivary glands over the number of positive- infected midguts at 7- and 14-dpve.

### BBKV transmission through mosquito saliva

**Fig 7:**
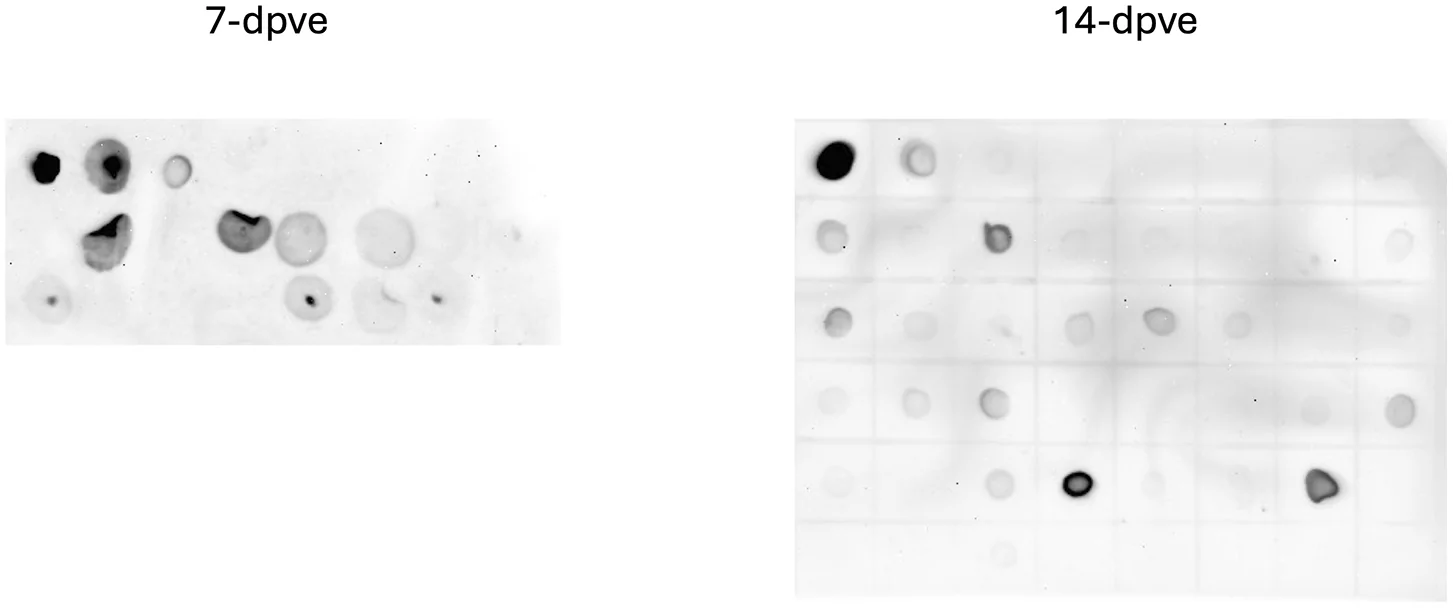
Detection of effective salivation on saliva samples collected. Saliva samples were deposited on PVDF membranes. After drying and blocking with a skimmed milk solution, the membranes were incubated in the presence of an anti-saliva polyclonal antibody and revealed by a peroxidase-labeled secondary antibody. The first row represents the positive controls with fold-dilution and negative controls and the rows below show results obtained with the samples.

Before detecting any viral protein in the saliva, the salivation needs to be confirmed. A first Dot blot on saliva samples was performed using polyclonal antibodies against the saliva protein of *Aedes* and *Culex* species as a primary antibody (Fig 7).

**Fig 8:**
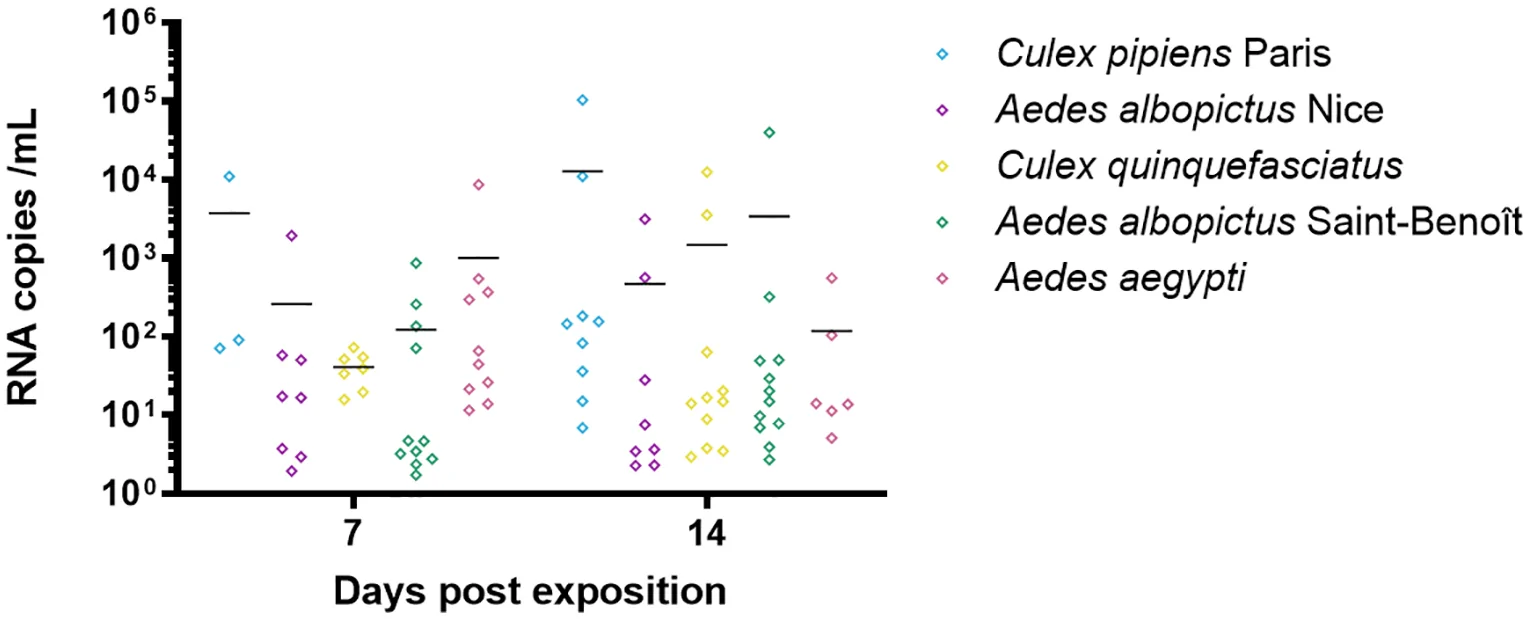
Viral RNA amplified for genome quantification by RT-qPCR in the saliva at 7- and 14-dpve. Saliva samples were collected at 7- and 14-dpve to BBKV. They were submitted to RNA extraction and RT-qPCR. Statistical tests were found not significative between the different species (Kruskal-Wallis test).

These results confirm effective salivation in most tested mosquitoes (S7 Fig). We then performed RT-qPCR on saliva samples (Fig 8). Viral RNA was detected in saliva samples at both 7- and 14-dpve (S10, S11 Tables).

Titration assays were performed to confirm if infectious viral particles were released in saliva. Viral titers ranging to 10^2^ to 10^3^ pfu were found in the saliva of *Aedes albopictus* and *Aedes aegypti* at D7 and D14 (S9 Table).

## Discussion

The present study aimed to assess the emergence potential of BBKV by evaluating its ability to infect, disseminate, and be transmitted by mosquito species from distinct ecological backgrounds. We provide direct experimental evidence that BBKV can efficiently complete its infection cycle in multiple mosquito species from both tropical and temperate regions. The detection of viral RNA in mosquito midgut, salivary glands and saliva demonstrates that the virus is able to overcome key vector barriers, supporting its capacity for transmission. Altogether, these findings indicate that BBKV is not restricted to a narrow vector range but instead exhibits broad vector competence and a strong potential for geographic expansion.

A key strength of this study lies in its ecologically grounded experimental design. Mosquito populations were maintained and tested at temperatures reflecting their natural environments, thereby preserving biologically relevant conditions under which vector competence is expressed. This approach does not aim to isolate intrinsic species-specific differences under artificial standardized conditions, but rather to evaluate the integrated performance of each species within its evolutionary thermal niche. Consequently, the observed differences reflect ecologically meaningful transmission capacities rather than methodological bias. This framework is particularly relevant for assessing emergence risk across climatic regions, as it identifies the mosquito species most likely to contribute to virus transmission under real-world conditions.

Biologically, the ability of BBKV to infect phylogenetically and ecologically distinct mosquito genera highlights a high level of viral plasticity, a feature commonly associated with arboviruses exhibiting strong emergence potential [24].

The involvement of both *Aedes* and *Culex* species is particularly informative, as these genera differ in their ecology, host preferences, and feeding behaviors, potentially facilitating the maintenance of the virus in distinct transmission cycles. Such flexibility may enable BBKV to bridge enzootic and more anthropophilic transmission patterns, as described for other alphaviruses [7,11,15]. In this context, the close phylogenetic relationship between BBKV and SINV, supported by high nucleotide identity and classification within the Western Equine Encephalitis antigenic complex [6,7], further suggests that BBKV may share similar transmission dynamics and adaptive potential. Among the tested species, *Aedes albopictus* emerged as the most efficient vector in both tropical and temperate settings, integrating high infection, dissemination, and transmission capacities.

Our results are consistent with previous field-based observations reporting the isolation of BBKV from a wide diversity of mosquito genera across sub-Saharan Africa, including *Anopheles*, *Aedes*, *Culex*, *Eretmapodites*, *Coquillettidia*, and *Mansonia* species [9–14]. However, while these studies demonstrated viral presence in natural settings, they did not provide direct evidence of vector competence. By experimentally demonstrating infection, dissemination, and potential transmission, our study extends these observations and provides functional validation of a broad vector range. This is in line with findings on related alphaviruses such as SINV, which are known for their ecological flexibility and ability to adapt to multiple vectors [7,15]. The high genetic similarity observed between BBKV and African SINV genotype I strains [6,7] further reinforces this parallel.

Some limitations should nevertheless be considered. Vector competence was assessed under controlled laboratory conditions, which do not fully capture the complexity of natural transmission cycles, including environmental variability and host availability. In addition, only a limited number of mosquito species and populations were tested, and intra-specific variability in susceptibility and transmission efficiency cannot be excluded. Finally, temperature, a key determinant of vector competence and arbovirus transmission dynamics, was incorporated here as an ecological parameter but not systematically dissected, despite its well-documented influence [25–27].

Taken together, our findings support the hypothesis that BBKV possesses a significant emergence potential. The identification of competent vectors in both tropical and temperate settings highlights its capacity to establish transmission cycles across diverse environments. Its ability to infect and be transmitted by mosquito species belonging to distinct ecological contexts suggests that it could spread beyond its currently recognized geographic range, particularly in the context of environmental change and vector expansion. Combined with previous reports of human infections and serological evidence of circulation [7,15], these results indicate that BBKV may be underrecognized and could represent an emerging arboviral threat.

In conclusion, BBKV emerges from this study as a potentially emerging arbovirus characterized by a broad ecological and vectorial niche. These findings underscore the need for enhanced surveillance and for integrative approaches combining vector competence, host susceptibility, and environmental drivers to better anticipate and mitigate its emergence.

## Supporting information

S1 Fig Western Blot Raw data to detect viral protein in midguts and salivary glands in *Aedes aegypti* PAEA at 7-dpve - chemiluminescent detection and white light exposure. (TIF)

S2 Fig Western Blot Raw data to detect viral protein in midguts and salivary glands in *Aedes aegypti* PAEA at 14-dpve - chemiluminescence detection and white light exposure. (TIF)

S3 Fig Western Blot Raw data to detect viral protein in midguts and salivary glands in *Culex pipiens molestus* PARIS at 7-dpve - chemiluminescence detection and white light exposure. (TIF)

S4 Fig Western Blot Raw data to detect viral protein in midguts and salivary glands in *Culex pipiens molestus* PARIS at 14-dpve - chemiluminescence detection and white light exposure. (TIF)

S5 Fig Western Blot Raw data to detect viral protein in midguts and salivary glands in *Culex quinquefasciatus* at 7-dpve - chemiluminescence detection and white light exposure. (TIF)

S6 Fig Western Blot Raw data to detect viral protein in midguts and salivary glands in *Culex quinquefasciatus* at 14-dpve - chemiluminescence detection and white light exposure. (TIF)

S7 Fig Dot Blot Raw data to detect viral protein in saliva in *Aedes aegypti* at 7-dpve - chemiluminescent detection and white light exposure. (TIF)

S1 Table Blood feeding rate for all mosquito species. (XLSX)

S2 Table Sample map of 384 well plates for RT-qPCR to quantify BBKV RNA. (XLSX)

S3 Table RTqPCR Raw data to quantify BBKV RNA in midguts, legs and salivary glands in *Aedes aegypti* PAEA at 7- and 14-dpve. (XLS)

S4 Table RTqPCR Raw data to quantify BBKV RNA in midguts, legs and salivary glands in *Aedes albopictus* NICE at 7- and 14-dpve. (XLS)

S5 Table RTqPCR Raw data to quantify BBKV RNA in midguts, legs and salivary glands in *Aedes albopictus* St Benoît at 7- and 14-dpve. (XLS)

S6 Table RTqPCR Raw data to quantify BBKV RNA in midguts, legs and salivary glands in *Culex quinquefasciatus* at 7- and 14-dpve. (XLS)

S7 Table RTqPCR Raw data to quantify BBKV RNA in midgut, legs and salivary glands for all species at 7- and 14-dpve. (XLSX)

S8 Table Western Blot Raw data to detect viral protein in midguts and salivary glands in *Aedes albopictus* NICE at 7- and 14-dpve and *Aedes albopictus* St Benoît at 14-dpve - chemiluminescence detection and white light exposure. (XLSX)

S9 Table Raw data titration plaque assay to quantify BBKV infectious particles in midguts and salivary glands at 7- and 14-dpve for all mosquito species. (XLSX)

S10 Table RT-qPCR Raw data to quantify BBKV RNA in saliva samples from all mosquito species #1. (XLS)

S11 Table RT-qPCR Raw data to quantify BBKV RNA in saliva samples from all mosquito species #2. (XLS)
