# Supplementary material for: Vector competence of temperate and tropical mosquito species for Babanki virus
Source: PLoS Negl Trop Dis. 2026 Aug 24;20(8):e0013051. doi: 10.1371/journal.pntd.0013051 (PMC13552956; doi:10.1371/journal.pntd.0013051)
Supplement: Supplementary file 1 [file pntd.0013051.s020.docx]

**Methods**

Reviewer #1: Yes, the study titled “Vector Competence of Temperate and Tropical Mosquito Species for Babanki Virus” by Mathilde Ban et al. (2025) presents research on vector competence using a clone of the Babanki virus (BBKV) and various mosquito species. The work features a robust and relevant experimental design and provides essential epidemiological information on BBKV. However, revisions are necessary. Populations of mosquitoes infected at different temperatures cannot be compared, as an increase in temperature may influence vector competence (28 °C for Culex quinquefasciatus and Aedes aegypti, and 24 °C for the other temperate species).

We thank the reviewer for this remark. It is correct that increasing the temperature may influence the vector competence for *Culex pipiens pipiens* (Voegels *et al.*, 2017; Jansen *et al.*; 2022) and *Aedes albopictus* (Gorreti Onyango *et al.*, 2019 ; Mercier *et al.*, 2022). For vector competence studies, incubation temperature is a critical determinant of the viral replication, extrinsic incubation period, and mosquito survival. Because mosquito populations originate from distinct climatic regions, their physiological optima and thermal tolerances differ substantially. Temperate mosquitoes, such as *Culex pipiens* or *Aedes albopictus* from northern latitudes, typically experience cooler environmental temperatures (15–25 °C) and have evolved life-history traits adapted to seasonal variability, diapause, and lower thermal maxima. In contrast, tropical species, including *Aedes aegypti* or *Culex quinquefasciatus*, develop and feed in consistently warmer climates (25–30 °C) and show maximal viral replication rates at higher temperatures. Therefore, incubation temperatures in laboratory experiments must reflect the thermal ecology of the mosquito population under study. Using temperatures appropriate for the species’ native environment ensures biologically realistic estimates of infection, dissemination, and transmission, while avoiding experimental bias caused by thermal stress or artificially accelerated viral growth. This approach also aligns with established vector-borne disease modeling frameworks, which recognize temperature as one of the strongest environmental drivers of vector competence.

More details have been added to the revised version of the manuscript lines 209-214.

**Results**

Reviewer #1: yes, the study addresses the dynamics of viral infection in a thorough and appropriate manner.

We thank the reviewer for this remark.

**Conclusions**

Reviewer #1: Yes, but it should be limited to the data from the study. Revisions are necessary.

We thank the reviewer for this remark. In this part, we are doing the discussion of the study.

**Summary and General Comments**

Reviewer #2: Dear authors, This is an interesting and well written research article dealing with the assessment of vector competence of tropical and temperate strains of Culex pipiens, Aedes albopictus, and Aedes aegypti for Babanki virus (BBKV). The introduction is relevant to the scope, the methodology and presentation of the results are quite clear, and the findings are very interesting. So, I have no major reservations for the manuscript and revisions to propose.

We thank the reviewer for this remark.

I would suggest starting the introduction with the second paragraph referring to BBKV and then continuing with the first paragraph which should be limited.

We thank the reviewer for this suggestion. Paragraphs were modified. Restriction of alphavirus introduction was limited because we think that it is interesting to understand why BBKV is relevant as an old world alphavirus genome that can be associated with new world alphavirus symptoms.

Lines 84-86: Could you please provide more information about the current knowledge for the transmission of BBKV to humans by mosquitoes?

Currently lines 66_68 in the revised version of the manuscript. We thank the reviewer for this comment. There is no current published article about the transmission of BBKV to humans by mosquitoes. There are only published articles about mosquito surveillance and serosurveillance. BBKV is closely related to SINV which is predominantly transmitted by *Culex* mosquitoes and occasionally *Aedes* species. BBKV is very likely transmitted through the same mosquito-borne pathway.

Line 286: Could you please clarify if these formulas derive from some specific literature reference? Normally, this should be the case, and the relevant reference should be added in the manuscript.
A significant amount of viral genome was found in the mosquito legs. Could you please discuss this finding a little bit? What is the importance and how is this interpreted?

We thank the reviewer for those comments. These formulas are from the paper of Danet *et al.*, 2019. It was added in the material and methods line 273 in the revised version of the manuscript.

The presence of the viral genome in the mosquito legs indicates that the virus was able to escape the infected midgut and disseminate to the legs where it replicates. Virus detection in mosquito legs is a standard indicator of systemic viral dissemination, as legs are among the first peripheral tissues infected once the midgut escape barrier is crossed. A high viral load in the legs suggests efficient viral replication and dissemination throughout the body. Although it does not directly indicate transmission competence—which is assessed in saliva—it reflects the mosquito’s ability to support systemic infection, a prerequisite for salivary gland invasion and transmission. We add in the discussion a short section esxplaining the meaning of the detection of viral genome in the mosquito legs lines 519-521 in the revised version of the manuscript.

Reviewer #3: Ban et al described a thorough vector competence study on Babanki virus, a closely related Sindbis virus. This study is important, and while the study was excellent and manuscript has been written in great details, some minor typo/mistakes remain.

We thank the reviewer for this remark.

Line 135 elaborate on c636 maintenance, why without CO2?

Now line 112 in the revised version of the manuscript. C6/36 can be cultivated in both conditions, with and without CO2. The one we had were cultivated without. To respect their culture’s condition, we followed these conditions.

Line 348 Any reasons to why the feeding exposure was differentiated?

Currently line 340 in the revised version of the manuscript*. Culex* preferred to feed on soak cotton whereas *Aedes* mosquitoes preferred to feed through a membrane.

Line 226 10% not italic

This has been corrected line 193 in the revised version of the manuscript.

Line 227 quinquefasciatus

This has been corrected line 212 in the revised version of the manuscript.

Line 233 Choose one “Samples collections/Mosquito dissection.”

This has been corrected line 215 in the revised version of the manuscript as “Mosquito sample dissection”

Line 233 “In the BSL-3 facility,” not bold

This has been corrected line 215 in the revised version of the manuscript.

Line 240 spell out abbr and product details of DMEM, SVF, P/S and ATB/ATF.

These abbreviations were first detailed in the Cells paragraph line 109-111. SVF is the French abbreviation of FBS which stand for fetal bovine serum. This has been corrected line 225 in the revised version of the manuscript.

Line 253 choose one ”Viral RNA quantification/RT-qPCR analysis.”

This has been corrected line 238 in the revised version of the manuscript as “Viral RNA quantification by RT-qPCR analysis”.

Line 258 describe in paragraph/description style; “Specific primers were used to amplify the viral RNA:

259 nsp2-Forward: AAGCGATGCGTTAAGAAGGA

260 nsp2-Reverse: ACTTGATGATAGCTGACTTGCC. “

This has been corrected lines 244-245 in the revised version of the manuscript.

Line 268 choose one is enough “Viral protein detection/Blot analysis.”

This has been corrected line 253 in the revised version of the manuscript as “Viral protein detection by Western Blot”

Line 268 “Western Blot.” “Dot blot” Not sure why bold words are in the middle of sentence. Authors may separate into two different paragraphs.

As suggested, two separated paragraphs were differentiated line 253 and 260 in the revised version of the manuscript.

Line 316, 321 unbold the words

This has been corrected in the revised version of the manuscript line 311 and 316.

Line 344 (p<0)?

Currently line 336 in the revised version of the manuscript, the p-value is <0.0001 which is very significative.

Line 354 why comparing 2 Culex species with Ae alb Nice which showed the lowest quantity. While the other two Aedes with quantity even higher than Culex were not described/emphasized.

No significative difference was observed between *Culex pipiens* Paris, C*x. quinquefasciatus* and *Aedes albopictus* Nice. However, in comparison to *Aedes albopictus* St-Benoit and *Ae. aegypti*, the number of RNA copies in their midgut is significantly lower (p<0.001). This has been detailed in the paragraph lines 345-352 in the revised version of the manuscript.

Reviewer #4: The authors present a vector competence study of temperate and tropical mosquitoes for Babanki virus. Although the study is technically sound and interesting, the whole document presents a lot of linguistic inaccuracies and missing information, e.g. it is completely unclear which Culex pipiens taxon (biotype?) is used in the experiments. The useage of any abbreviations including mosquito genera is not uniform.

We thank the reviewer for this comment. We used *Culex pipiens molestus* that will then be referred as *Culex pipiens* Paris in the rest of the manuscript. We abbreviated *Culex* as *Cx.* and *Aedes* as *Ae. w*hen they are not in the beginning of the sentence in the revised version of the manuscript.

I really miss the reasoning for the study, i.e. how large is the probability of spread of the virus and how could this happen?

We thank the reviewer for this remark. The aim of this study is to characterize BBKV vector competence to analyze the potential emergence of BBKV to new tropical or temperate area. No probability was performed regarding the spread of the virus. For example, if we compare with dengue virus infection, before in Europe there were no *Aedes albopictus* so no vector of the virus. Now *Ae. albopictus* is established in more than 15 European countries. Autochthonous dengue virus infection cases in Europe are increasing and becoming a public health interest in temperate areas.

The discussion is a lot of repetition of the results without discussion or with missing references.

We thank the reviewer for this comment. Result repetitions were deleted in the revised version of the manuscript.

55: What do you mean by preferred condition? Does a virus prefer something?

57: known vector

58: host-range varies in space, time, between countries, between viruses?

In this part, authors wanted to introduced Alphavirus in general. Yes, alphavirus can be geographically restricted by the area temperature or host. (Kraemer *et al.*, 2015 ; Sahadeao *et al.*; 2017 ; Phadungsombat *et al.*, 2022 ; Hayes *et al.*, 1976 ; Lwande *et al.*, 2013).

64: introduce abbreviation of SINV already here

This has been corrected line 78 in the revised version of the manuscript.

64: chikungunya virus not Chikungunya virus

This has been corrected line 79 in the revised version of the manuscript.

84-85: what is ArboCat? Did the virus really spread or is it just a surveillance artefact?

Currently line 59-60 in the revised version of the manuscript. Arbocat is the Arbovirus Catalogue that is maintain by Centers for Disease Control and Prevention from the United States of America.

93: please abbreviate mosquito genera after introduction, check the manuscript

This has been corrected line 89 in the revised version of the manuscript.

105: which Culex species?

We are describing the mosquito species in the paragraph lines 177-184 in the revised version of the manuscript. In our study we tested *Culex pipiens molestus* and *Culex quinquefasciatus*.

121: For the emergence there should be also some ideas on how the virus could spread to other regions…
123: which regions?

Currently paragraph lines 101-106 in the revised version of the manuscript. BBKV is closely related to SINV and is found in mosquitoes that are feeding on both humans and birds, the virus could spread to other region through migratory birds (Ling et al., 2019).

147: E. coli does not have to be introduced as abbreviation

This has been corrected lines 124-125 in the revised version of the manuscript.

199: please use Culex pipiens biotype pipiens or which kind of mosquito did you use? Completely unclear

This has been corrected line 180 in the revised version of the manuscript. *Culex pipiens molestus* were used in this study. It could be referred as “*Culex pipiens* Paris” in the remainder of the manuscript.

226: 10% not in italics

This has been corrected line 193 in the revised version of the manuscript.

230-252: Which specimens are fully dissected, and for which do you collect saliva, or do you just present the techniques in the wrong temporal order?

Currently paragraphs lines 215-237 in the revised version of the manuscript. There are 2 different specimens used for sample collection. A first mosquito specimen was used to its midgut, legs and salivary glands. A second specimen of mosquito was used to collect only its saliva.

300: Was the abbreviation of E. coli not unessarily introduced before?

This has been corrected line 295 in the revised version of the manuscript.

307: NNPP is a unnecessary abbreviation as only used once

NNPP is first used in the material in line 123. Then is the results part in lines 296, 301 and 326.

520-540: This is not a discussion of the results and belongs to the introduction or methods
528: Do not abbreviate the genus at the start of the sentence

Previous paragraph lines 520-526 was added to the current Results part lines 284-289 in the revised version of the manuscript.

Previous lines 527 to 540 are still in the discussion (501-506 and 525-532). We are discussing our study results with the literature.

There is no more abbreviation of the genus at the start of the sentence in the revised version of the manuscript.

572-: Here and the following sentences references are missing.

References about SINV and BBKV infected *Culex* species has been added line 538 in the revised version of the manuscript.

572-: I miss a discussion of the age of the mosquito colonies? Can they have a negative impact?

A short discussion about the age of the mosquito colonies was added lines 545-549 in the revised version of the manuscript. The age of the mosquito colonies can substantially influence vector competence outcomes. Long established laboratory colonies undergo genetic drift, inbreeding and adaptation to stable rearing conditions, which may alter traits such as immunity, microbiota composition feeding behavior, and midgut barrier integrity. In contrast, recent field-derived populations retain natural genetic diversity and ecological adaptations shaped by fluctuating environmental pressures. These differences can lead to variations in infection, dissemination and transmission rates. It would be an interesting study to be able to compare recent field-derived and long-established laboratory mosquito from the same species and genus.
