# Supplementary material for: Vector competence of temperate and tropical mosquito species for Babanki virus
Source: PLoS Negl Trop Dis. 2026 Aug 24;20(8):e0013051. doi: 10.1371/journal.pntd.0013051 (PMC13552956; doi:10.1371/journal.pntd.0013051)
Supplement: Supplementary file 2 [file pntd.0013051.s021.docx]

**Response to Reviewers**

**Summary and General Comments**

Use this section to provide overall comments, discuss strengths/weaknesses of the study, novelty, significance, general execution and scholarship. You may also include additional comments for the author, including concerns about dual publication, research ethics, or publication ethics. If requesting major revision, please articulate the new experiments that are needed.

Reviewer#1: PLOS Neglected Tropical Diseases
Vector Competence of Temperate and Tropical Mosquito Species for Babanki virus

Introduction
The first paragraph is appropriate and well aligned with the introduction. However, the second and third paragraphs appear weakly connected to the main problem and the objective of the study, which disrupts the logical flow of the introduction. It is not entirely clear how this information contributes to understanding the study. In addition, the objective is introduced without a clear transition linking it to the preceding sections.

Thank you for this comment.

The introduction was previously modified thanks to reviewer #2 comments. It was changed again according to your comments, increasing readability and fluidity.

Methods
The methodology is comprehensive and ambitious; however, the statistical analysis is not appropriate for the interpretation of the data presented.

Results
My previous suggestion was not addressed. I agree that each mosquito species has its own optimal rearing temperature and that, for vector competence assays to be ecologically relevant, laboratory conditions should reflect those of the environments in which the species naturally breed. However, the main flaw of the manuscript is the inclusion of temperature as a confounding factor. The authors compare species maintained at different temperatures, for example Cx. quinquefasciatus, Ae. aegypti, and Ae. albopictus St-Benoît (28 °C) versus Cx. pipiens molestus Paris and Ae. albopictus Nice (24 °C). The statistical analysis is technically appropriate for simple group comparisons but is not suitable for supporting interspecific comparisons when species were evaluated at different temperatures. Ideally, an analytical approach that incorporates temperature as a factor (e.g., generalized models) should be used. Temperature is a well-known factor that directly affects vector competence and therefore cannot be considered a neutral variable. Because temperature was not controlled, the observed differences cannot be attributed exclusively to species effects.

We thank the reviewer for his careful reading of our manuscript and for raising this important point regarding the relationship between species identity and ecological evolution temperature. Statistical analyses were reviewed in consultation with a biostatistician from the Bioinformatics and Biostatistics Hub at Institut Pasteur, who is now among the authors of our manuscript (Emeline Perthame). We carefully considered the suggestion to normalize the data by temperature prior to comparison; however, we respectfully disagree with this approach based on the specific objectives and design of our study.

Our experimental design was constructed to evaluate species performance under their respective native thermal regimes (i.e., **“in situ”** conditions), rather than to isolate the plastic response of a single species across a thermal gradient. In this context, temperature is not a nuisance variable or a confounder to be removed; it is an intrinsic component of the ecological niche we are comparing.

Statistically, "normalizing" the data by temperature would effectively remove the variance attributable to the interaction between species identity and their adapted thermal environment. This would obscure the very biological phenomenon we aim to quantify: how different species perform under the specific conditions they encounter in the wild? If we were to normalize across temperatures, we would be comparing hypothetical, non-ecological scenarios rather than the observed reality of these populations.

We nonetheless followed the advice of the reviewer and tested a linear mixed model. For this purpose, RT-qPCR and PFU titers results were log-transformed to meet linear model assumptions of normality and homoscedasticity. A mixed linear model was fitted using the lmer function of the lme4 R package, with log-transformed titers as the response variable, species, days, and their interaction as fixed effects and the temperature as random intercept. Model fit and assumptions were assessed using diagnostic plots generated by the check_model function of the performance R package [Lüdecke et al., (2021). performance: An R Package for Assessment, Comparison and Testing of Statistical Models. Journal of Open Source Software, 6(60), 3139. https://doi.org/10.21105/joss.03139]. Estimated marginal means and pairwise comparisons between species within each day were computed with the emmeans package [Lenth R, Piaskowski J (2026). emmeans: Estimated Marginal Means, aka Least-Squares Means. doi:10.32614/CRAN.package.emmeans <https://doi.org/10.32614/CRAN.package.emmeans>, R package version 2.0.2, <https://CRAN.R-project.org/package=emmeans>.], and p-values for multiple comparisons were adjusted using Tukey’s method.

|  |  | QPCR | | |
| --- | --- | --- | --- | --- |
|  |  | MG | SG | Legs |
| Comparison | days | p.value | p.value | p.value |
| Aedes albopictus NICE-Aedes albopictus Saint-Benoît | 7 | 0,32461968 | 0,71998772 | 0,56513249 |
| Aedes albopictus NICE-Culex quinquefasciatus | 7 | 0,99947933 | 0,34560175 | 0,88664824 |
| Aedes albopictus NICE-Aedes aegypti PAEA | 7 | 0,45786001 | 0,21249368 | 0,13225609 |
| Aedes albopictus NICE-Culex pipiens PARIS | 7 | 0,99997004 | 0,0040187 | 0,99976903 |
| Aedes albopictus Saint-Benoît-Culex quinquefasciatus | 7 | 0,0036482 | 0,85246853 | 0,91676716 |
| Aedes albopictus Saint-Benoît-Aedes aegypti PAEA | 7 | 0,97270597 | 0,61646048 | 0,73725782 |
| Aedes albopictus Saint-Benoît-Culex pipiens PARIS | 7 | 0,36667694 | 0,93863297 | 0,66874936 |
| Culex quinquefasciatus-Aedes aegypti PAEA | 7 | 0,00920297 | 0,99974741 | 0,20346746 |
| Culex quinquefasciatus-Culex pipiens PARIS | 7 | 0,99979804 | 0,99999144 | 0,93445103 |
| Aedes aegypti PAEA-Culex pipiens PARIS | 7 | 0,50435564 | 0,9999994 | 0,20344455 |
| Aedes albopictus NICE-Aedes albopictus Saint-Benoît | 14 | 0,2905044 | 0,08959829 | 0,28708107 |
| Aedes albopictus NICE-Culex quinquefasciatus | 14 | 0,99996662 | 0,71535468 | 0,99723345 |
| Aedes albopictus NICE-Aedes aegypti PAEA | 14 | 0,20267806 | 0,21061046 | 0,11842929 |
| Aedes albopictus NICE-Culex pipiens PARIS | 14 | 0,5180707 | 0,02362486 | 0,09749852 |
| Aedes albopictus Saint-Benoît-Culex quinquefasciatus | 14 | 5,9622E-06 | 0,2736432 | 0,0565465 |
| Aedes albopictus Saint-Benoît-Aedes aegypti PAEA | 14 | 0,99381328 | 0,96464675 | 0,97562134 |
| Aedes albopictus Saint-Benoît-Culex pipiens PARIS | 14 | 0,69859214 | 0,99163041 | 0,99537025 |
| Culex quinquefasciatus-Aedes aegypti PAEA | 14 | 3,9513E-07 | 0,64521817 | 0,00539029 |
| Culex quinquefasciatus-Culex pipiens PARIS | 14 | 0,94551819 | 0,96404984 | 0,7333373 |
| Aedes aegypti PAEA-Culex pipiens PARIS | 14 | 0,58006487 | 0,99998304 | 0,93801686 |

As expected by adding the temperature as a random effect, only comparisons of species of the same temperature condition were significative.

While we acknowledge that species and temperature are collinear in this specific subset of the data, this collinearity is a feature of the natural system we are studying, not an experimental artifact. Our analysis focuses on the **“observed marginal means”** of each species under its specific ecological evolution condition, which provides a valid comparison of ecological fitness across these distinct groups. Introducing a statistical correction to decouple temperature from species would result in an over-correction that eliminates the biological signal of local adaptation.

In response to the reviewer’s comment, we have clarified this point in both the Abstract, Methods, Results and Discussion sections to avoid overinterpretation. Specifically, we now emphasize that the observed differences between mosquito species should be interpreted within their respective ecological contexts rather than as direct intrinsic comparisons. We explicitly clarify that our comparisons reflect performance under native thermal conditions and that temperature was treated as a fixed effect inherent to the species' origin rather than a covariate to be normalized.

We have also refined the wording throughout the manuscript to ensure a more cautious interpretation of the data. Statements suggesting persistence or transmission efficiency have been adjusted to remain strictly supported by experimental evidence.

These modifications improve the clarity and robustness of the interpretation while preserving the original structure and results of the manuscript.

Moreover, following statistical review, analyses were performed using non-parametric methods. Differences between groups were assessed using the Kruskal–Wallis test followed by post hoc multiple comparisons with Tukey-type correction, implemented in Simstat software (v2.5.8). Parametric analyses (two-way ANOVA) initially performed in Prism were not retained, as non-parametric approaches were considered more appropriate for the distribution and structure of the data in this study.

Lines 382-383: This phrase should go in the discussion section, not here. “In conclusion, BBKV replicated better in the midguts of Aedes species than in those of Culex species”.

Thank you for this comment, the sentence was removed.

Discussion
The discussion does not follow a clear hierarchical structure, for example: (i) main findings, (ii) biological implications, (iii) comparison with previous studies, (iv) limitations, and (v) epidemiological consequences. Instead, topics are interspersed, which disrupts the narrative flow. The discussion does not begin with the most relevant result, such as the efficient infection and dissemination of BBKV in multiple mosquito species or its broad vector range across Aedes and Culex. The paragraphs are weakly connected, and there is no clear guiding thread linking the results to the stated objective of the study. The first paragraph reiterates epidemiological background information (historical isolation, serology, underdiagnosis), which belongs in the Introduction rather than the Discussion. From the second/third paragraph onward, although valid results are discussed, each topic appears almost as an independent block. As a consequence, the main objective of the study (emergence potential) becomes diluted, and the discussion remains largely descriptive rather than interpretative and synthetic.

We thank the reviewer for this important and constructive comment. We agree that the original Discussion lacked a clear hierarchical structure and that the narrative flow could be improved.

To address this issue, we have thoroughly reorganized the Discussion following a structured framework: (i) main findings, (ii) biological implications, (iii) comparison with previous studies, (iv) limitations, and (v) epidemiological consequences. We also removed redundant epidemiological background information from the opening paragraph and refocused the Discussion on interpretation rather than description.

Importantly, the revised version now begins with the most relevant findings of the study—namely the efficient infection, dissemination, and transmission of BBKV across multiple mosquito species from both *Aedes* and *Culex* genera—and consistently links these results to the central objective of assessing the emergence potential of BBKV.

These changes significantly improve the clarity, coherence, and scientific impact of the Discussion.
