# Supplementary material for: Vector competence of temperate and tropical mosquito species for Babanki virus
Source: PLoS Negl Trop Dis. 2026 Aug 24;20(8):e0013051. doi: 10.1371/journal.pntd.0013051 (PMC13552956; doi:10.1371/journal.pntd.0013051)
Supplement: Peer review history

# Peer review: Decision Letter 0

**DOI:** 10.1371/journal.pntd.0013051.r001

**Authors:** Abdallah M. Samy

12 Sep 2025

PNTD-D-25-00563

Vector Competence of Temperate and Tropical Mosquito Species for Babanki virus

PLOS Neglected Tropical Diseases

Dear Dr. Choumet,

Thank you for submitting your manuscript to PLOS Neglected Tropical Diseases. After careful consideration, we feel that it has merit but does not fully meet PLOS Neglected Tropical Diseases's publication criteria as it currently stands. Therefore, we invite you to submit a revised version of the manuscript that addresses the points raised during the review process.

Please submit your revised manuscript within 60 days Nov 11 2025 11:59PM. If you will need more time than this to complete your revisions, please reply to this message or contact the journal office at plosntds@plos.org. When you're ready to submit your revision, log on to https://www.editorialmanager.com/pntd/ and select the 'Submissions Needing Revision' folder to locate your manuscript file.

Please include the following items when submitting your revised manuscript:

* A rebuttal letter that responds to each point raised by the editor and reviewer(s). You should upload this letter as a separate file labeled 'Response to Reviewers'. This file does not need to include responses to any formatting updates and technical items listed in the 'Journal Requirements' section below.

* A marked-up copy of your manuscript that highlights changes made to the original version. You should upload this as a separate file labeled 'Revised Manuscript with Track Changes'.

* An unmarked version of your revised paper without tracked changes. You should upload this as a separate file labeled 'Manuscript'.

If you would like to make changes to your financial disclosure, competing interests statement, or data availability statement, please make these updates within the submission form at the time of resubmission. Guidelines for resubmitting your figure files are available below the reviewer comments at the end of this letter.

We look forward to receiving your revised manuscript.

Kind regards,

Abdallah Samy

Section Editor

PLOS Neglected Tropical Diseases

Shaden Kamhawi

co-Editor-in-Chief

PLOS Neglected Tropical Diseases

orcid.org/0000-0003-4304-636XX

Paul Brindley

co-Editor-in-Chief

PLOS Neglected Tropical Diseases

orcid.org/0000-0003-1765-0002

**Additional Editor Comments:**

We have received four reviews of your manuscript. The referees have raised important concerns that must be addressed before the paper can be reconsidered. Please submit a revised manuscript that responds to each reviewer comment in a point-by-point response letter, indicating where changes were made (include line numbers) or providing a reasoned justification where you disagree. Provide both a clean version of the revised manuscript and a tracked-changes version highlighting edits. We look forward to receiving your revision.

**Journal Requirements:**

1) Please ensure that the CRediT author contributions listed for every co-author are completed accurately and in full.

At this stage, the following Authors/Authors require contributions: Mathilde Ban, Virginie Geolier, Elisabeth Ferquel, Martin Faye, Moussa Moise Diagne, El Hadji Ndiaye, Gamou Fall, Mawlouth Diallo, and Dimitri Lavillette. Please ensure that the full contributions of each author are acknowledged in the "Add/Edit/Remove Authors" section of our submission form.

The list of CRediT author contributions may be found here: https://journals.plos.org/plosntds/s/authorship#loc-author-contributions

2) We noticed that you used the phrase 'data not shown' in the manuscript. We do not allow these references, as the PLOS data access policy requires that all data be either published with the manuscript or made available in a publicly accessible database. Please amend the supplementary material to include the referenced data or remove the references.

3) We do not publish any copyright or trademark symbols that usually accompany proprietary names, eg ©,  ®, or TM  (e.g. next to drug or reagent names). Therefore please remove all instances of trademark/copyright symbols throughout the text, including:

- ® on page: 7 and 11.

- TM on pages: 7, 11, and 12.

4) We have noticed that you have uploaded Supporting Information files, but you have not included a list of legends. Please add a full list of legends for your Supporting Information files after the references list.

5) In the online submission form, you indicated that "data will be fully available". All PLOS journals now require all data underlying the findings described in their manuscript to be freely available to other researchers, either

- In a public repository

- Within the manuscript itself

- Uploaded as supplementary information.

This policy applies to all data except where public deposition would breach compliance with the protocol approved by your research ethics board. If your data cannot be made publicly available for ethical or legal reasons (e.g., public availability would compromise patient privacy), please explain your reasons by return email and your exemption request will be escalated to the editor for approval. Your exemption request will be handled independently and will not hold up the peer review process, but will need to be resolved should your manuscript be accepted for publication. One of the Editorial team will then be in touch if there are any issues.

**Reviewers' Comments:**

Reviewer's Responses to Questions

**Key Review Criteria Required for Acceptance?**

As you describe the new analyses required for acceptance, please consider the following:

**Methods**

-Are the objectives of the study clearly articulated with a clear testable hypothesis stated?

-Is the study design appropriate to address the stated objectives?

-Is the population clearly described and appropriate for the hypothesis being tested?

-Is the sample size sufficient to ensure adequate power to address the hypothesis being tested?

-Were correct statistical analysis used to support conclusions?

-Are there concerns about ethical or regulatory requirements being met?

Reviewer #1: Yes, the study titled “Vector Competence of Temperate and Tropical Mosquito Species for Babanki Virus” by Mathilde Ban et al. (2025) presents research on vector competence using a clone of the Babanki virus (BBKV) and various mosquito species. The work features a robust and relevant experimental design and provides essential epidemiological information on BBKV. However, revisions are necessary. Populations of mosquitoes infected at different temperatures cannot be compared, as an increase in temperature may influence vector competence (28 °C for Culex quinquefasciatus and Aedes aegypti, and 24 °C for the other temperate species).

Reviewer #2: (No Response)

Reviewer #3: (No Response)

Reviewer #4: (No Response)

**********

**Results**

-Does the analysis presented match the analysis plan?

-Are the results clearly and completely presented?

-Are the figures (Tables, Images) of sufficient quality for clarity?

Reviewer #1: yes, the study addresses the dynamics of viral infection in a thorough and appropriate manner.

Reviewer #2: (No Response)

Reviewer #3: (No Response)

Reviewer #4: (No Response)

**********

**Conclusions**

-Are the conclusions supported by the data presented?

-Are the limitations of analysis clearly described?

-Do the authors discuss how these data can be helpful to advance our understanding of the topic under study?

-Is public health relevance addressed?

Reviewer #1: Yes, but it should be limited to the data from the study. Revisions are necessary.

Reviewer #2: (No Response)

Reviewer #3: (No Response)

Reviewer #4: (No Response)

**********

**Editorial and Data Presentation Modifications?**

Use this section for editorial suggestions as well as relatively minor modifications of existing data that would enhance clarity. If the only modifications needed are minor and/or editorial, you may wish to recommend “Minor Revision” or “Accept”.

Reviewer #1: Major revision

Reviewer #2: (No Response)

Reviewer #3: (No Response)

Reviewer #4: (No Response)

**********

**Summary and General Comments**

Use this section to provide overall comments, discuss strengths/weaknesses of the study, novelty, significance, general execution and scholarship. You may also include additional comments for the author, including concerns about dual publication, research ethics, or publication ethics. If requesting major revision, please articulate the new experiments that are needed.

Reviewer #1: (No Response)

Reviewer #2: Dear authors,

This is an interesting and well written research article dealing with the assessment of vector competence of tropical and temperate strains of Culex pipiens, Aedes albopictus, and Aedes aegypti for Babanki virus (BBKV).

The introduction is relevant to the scope, the methodology and presentation of the results are quite clear, and the findings are very interesting. So, I have no major reservations for the manuscript and revisions to propose.

I would suggest starting the introduction with the second paragraph referring to BBKV and then continuing with the first paragraph which should be limited.

Lines 84-86: Could you please provide more information about the current knowledge for the transmission of BBKV to humans by mosquitoes?

Line 286: Could you please clarify if these formulas derive from some specific literature reference? Normally, this should be the case, and the relevant reference should be added in the manuscript.

A significant amount of viral genome was found in the mosquito legs. Could you please discuss this finding a little bit? What is the importance and how is this interpreted?

Reviewer #3: Ban et al described a thorough vector competence study on Babanki virus, a closely related Sindbis virus. This study is important, and while the study was excellent and manuscript has been written in great details, some minor typo/mistakes remain.

Line 135 elaborate on c636 maintenance, why without CO2?

Line 348 Any reasons to why the feeding exposure was differentiated?

Line 226 10% not italic

Line 227 quinquefasciatus

Line 233 Choose one “Samples collections/Mosquito dissection.”

Line 233 “In the BSL-3 facility,” not bold

Line 240 spell out abbr and product details of DMEM, SVF, P/S and ATB/ATF.

Line 253 choose one ”Viral RNA quantification/RT-qPCR analysis.”

Line 258 describe in paragraph/description style; “Specific primers were used to amplify the viral RNA:

259 nsp2-Forward: AAGCGATGCGTTAAGAAGGA

260 nsp2-Reverse: ACTTGATGATAGCTGACTTGCC. “

Line 268 choose one is enough “Viral protein detection/Blot analysis.”

Line 268 “Western Blot.” “Dot blot” Not sure why bold words are in the middle of sentence. Authors may separate into two different paragraphs.

Line 316, 321 unbold the words

Line 344 (p<0)?

Line 354 why comparing 2 Culex species with Ae alb Nice which showed the lowest quantity. While the other two Aedes with quantity even higher than Culex were not described/emphasized.

Reviewer #4: The authors present a vector competence study of temperate and tropical mosquitoes for Babanki virus. Although the study is technically sound and interesting, the whole document presents a lot of linguistic inaccuracies and missing information, e.g. it is completely unclear which Culex pipiens taxon (biotype?) is used in the experiments. The useage of any abbreviations including mosquito genera is not uniform.

I really miss the reasoning for the study, i.e. how large is the probability of spread of the virus and how could this happen?

The discussion is a lot of repetition of the results without discussion or with missing references.

55: What do you mean by preferred condition? Does a virus prefer something?

57: known vectors

58: host-range varies in space, time, between countries, between viruses?

64: introduce abbreviation of SINV already here

64: chikungunya virus not Chikungunya virus

84-85: what is ArboCat? Did the virus really spread or is it just a surveillance artefact?

93: please abbreviate mosquito genera after introduction, check the manuscript

105: which Culex species?

121: For the emergence there should be also some ideas on how the virus could spread to other regions…

123: which regions?

147: E. coli does not have to be introduced as abbreviation

199: please use Culex pipiens biotype pipiens or which kind of mosquito did you use? Completely unclear

226: 10% not in italics

230-252: Which specimens are fully dissected, and for which do you collect saliva, or do you just present the techniques in the wrong temporal order?

300: Was the abbreviation of E. coli not unessarily introduced before?

307: NNPP is a unnecessary abbreviation as only used once

520-540: This is not a discussion of the results and belongs to the introduction or methods

528: Do not abbreviate the genus at the start of the sentence

572-: Here and the following sentences references are missing.

572-: I miss a discussion of the age of the mosquito colonies? Can they have a negative impact?

**********

PLOS authors have the option to publish the peer review history of their article (what does this mean?). If published, this will include your full peer review and any attached files.

If you choose “no”, your identity will remain anonymous but your review may still be made public.

**Do you want your identity to be public for this peer review?**  For information about this choice, including consent withdrawal, please see our Privacy Policy.

Reviewer #1: No

Reviewer #2: No

Reviewer #3: **Yes:** Astri Nur Faizah

Reviewer #4: No

[NOTE: If reviewer comments were submitted as an attachment file, they will be attached to this email and accessible via the submission site. Please log into your account, locate the manuscript record, and check for the action link "View Attachments". If this link does not appear, there are no attachment files.]

**Figure resubmission:** While revising your submission, we strongly recommend that you use PLOS’s NAAS tool (https://ngplosjournals.pagemajik.ai/artanalysis) to test your figure files. NAAS can convert your figure files to the TIFF file type and meet basic requirements (such as print size, resolution), or provide you with a report on issues that do not meet our requirements and that NAAS cannot fix.

After uploading your figures to PLOS’s NAAS tool - https://ngplosjournals.pagemajik.ai/artanalysis, NAAS will process the files provided and display the results in the "Uploaded Files" section of the page as the processing is complete. If the uploaded figures meet our requirements (or NAAS is able to fix the files to meet our requirements), the figure will be marked as "fixed" above. If NAAS is unable to fix the files, a red "failed" label will appear above. When NAAS has confirmed that the figure files meet our requirements, please download the file via the download option, and include these NAAS processed figure files when submitting your revised manuscript.

**Reproducibility:**

To enhance the reproducibility of your results, we recommend that authors of applicable studies deposit laboratory protocols in protocols.io, where a protocol can be assigned its own identifier (DOI) such that it can be cited independently in the future. Additionally, PLOS ONE offers an option to publish peer-reviewed clinical study protocols. Read more information on sharing protocols at https://plos.org/protocols?utm_medium=editorial-email&utm_source=authorletters&utm_campaign=protocols

# Author comment: Author response to Decision Letter 1

**DOI:** 10.1371/journal.pntd.0013051.r002

31 Dec 2025

Attachment Submitted filename: response_letter.docx

# Peer review: Decision Letter 1

**DOI:** 10.1371/journal.pntd.0013051.r003

**Authors:** Abdallah M. Samy

19 Mar 2026

PNTD-D-25-00563R1

Vector Competence of Temperate and Tropical Mosquito Species for Babanki virus

PLOS Neglected Tropical Diseases

Dear Dr. Choumet,

Thank you for submitting your manuscript to PLOS Neglected Tropical Diseases. After careful consideration, we feel that it has merit but does not fully meet PLOS Neglected Tropical Diseases's publication criteria as it currently stands. Therefore, we invite you to submit a revised version of the manuscript that addresses the points raised during the review process.

Please submit your revised manuscript within by Apr 18 2026 11:59PM. If you will need more time than this to complete your revisions, please reply to this message or contact the journal office at plosntds@plos.org. When you're ready to submit your revision, log on to https://www.editorialmanager.com/pntd/ and select the 'Submissions Needing Revision' folder to locate your manuscript file.

Please include the following items when submitting your revised manuscript:

* A letter that responds to each point raised by the editor and reviewer(s). You should upload this letter as a separate file labeled 'Response to Reviewers'. This file does not need to include responses to any formatting updates and technical items listed in the 'Journal Requirements' section below.

* A marked-up copy of your manuscript that highlights changes made to the original version. You should upload this as a separate file labeled 'Revised Manuscript with Track Changes'.

* An unmarked version of your revised paper without tracked changes. You should upload this as a separate file labeled 'Manuscript'.

If you would like to make changes to your financial disclosure, competing interests statement, or data availability statement, please make these updates within the submission form at the time of resubmission. Guidelines for resubmitting your figure files are available below the reviewer comments at the end of this letter.

We look forward to receiving your revised manuscript.

Kind regards,

Abdallah M. Samy, PhD

Section Editor

PLOS Neglected Tropical Diseases

Abdallah Samy

Section Editor

PLOS Neglected Tropical Diseases

Shaden Kamhawi

co-Editor-in-Chief

PLOS Neglected Tropical Diseases

orcid.org/0000-0003-4304-636XX

Paul Brindley

co-Editor-in-Chief

PLOS Neglected Tropical Diseases

orcid.org/0000-0003-1765-0002

**Additional Editor Comments:**

Please consider carefully the reviewer comments below. The main points raised by one of the reviews are still important to be addressed before considering a decision on your manuscript.

**Journal Requirements:**

If the reviewer comments include a recommendation to cite specific previously published works, please review and evaluate these publications to determine whether they are relevant and should be cited. There is no requirement to cite these works unless the editor has indicated otherwise.

1) Please ensure that the CRediT author contributions listed for every co-author are completed accurately and in full.

At this stage, the following Authors/Authors require contributions: Dimitri Lavillette. Please ensure that the full contributions of each author are acknowledged in the "Add/Edit/Remove Authors" section of our submission form.

The list of CRediT author contributions may be found here: https://journals.plos.org/plosntds/s/authorship#loc-author-contributions

**Reviewers' Comments:**

Reviewer's Responses to Questions

**Key Review Criteria Required for Acceptance?**

As you describe the new analyses required for acceptance, please consider the following:

**Methods**

-Are the objectives of the study clearly articulated with a clear testable hypothesis stated?

-Is the study design appropriate to address the stated objectives?

-Is the population clearly described and appropriate for the hypothesis being tested?

-Is the sample size sufficient to ensure adequate power to address the hypothesis being tested?

-Were correct statistical analysis used to support conclusions?

-Are there concerns about ethical or regulatory requirements being met?

Reviewer #1: (No Response)

Reviewer #2: The methods are adequately presented.

Reviewer #3: (No Response)

**********

**Results**

-Does the analysis presented match the analysis plan?

-Are the results clearly and completely presented?

-Are the figures (Tables, Images) of sufficient quality for clarity?

Reviewer #1: (No Response)

Reviewer #2: The results are adequately presented.

Reviewer #3: (No Response)

**********

**Conclusions**

-Are the conclusions supported by the data presented?

-Are the limitations of analysis clearly described?

-Do the authors discuss how these data can be helpful to advance our understanding of the topic under study?

-Is public health relevance addressed?

Reviewer #1: (No Response)

Reviewer #2: The conclusions are adequately supported by the results.

Reviewer #3: (No Response)

**********

**Editorial and Data Presentation Modifications?**

Use this section for editorial suggestions as well as relatively minor modifications of existing data that would enhance clarity. If the only modifications needed are minor and/or editorial, you may wish to recommend “Minor Revision” or “Accept”.

Reviewer #1: (No Response)

Reviewer #2: I have no further comments.

Reviewer #3: (No Response)

**********

**Summary and General Comments**

Use this section to provide overall comments, discuss strengths/weaknesses of the study, novelty, significance, general execution and scholarship. You may also include additional comments for the author, including concerns about dual publication, research ethics, or publication ethics. If requesting major revision, please articulate the new experiments that are needed.

Reviewer #1: PLOS Neglected Tropical Diseases

Vector Competence of Temperate and Tropical Mosquito Species for Babanki virus

Introduction

The first paragraph is appropriate and well aligned with the introduction. However, the second and third paragraphs appear weakly connected to the main problem and the objective of the study, which disrupts the logical flow of the introduction. It is not entirely clear how this information contributes to understanding the study. In addition, the objective is introduced without a clear transition linking it to the preceding sections.

Methods

The methodology is comprehensive and ambitious; however, the statistical analysis is not appropriate for the interpretation of the data presented.

Results

My previous suggestion was not addressed. I agree that each mosquito species has its own optimal rearing temperature and that, for vector competence assays to be ecologically relevant, laboratory conditions should reflect those of the environments in which the species naturally breed. However, the main flaw of the manuscript is the inclusion of temperature as a confounding factor. The authors compare species maintained at different temperatures, for example Cx. quinquefasciatus, Ae. aegypti, and Ae. albopictus St-Benoît (28 °C) versus Cx. pipiens molestus Paris and Ae. albopictus Nice (24 °C). The statistical analysis is technically appropriate for simple group comparisons but is not suitable for supporting interspecific comparisons when species were evaluated at different temperatures. Ideally, an analytical approach that incorporates temperature as a factor (e.g., generalized models) should be used. Temperature is a well-known factor that directly affects vector competence and therefore cannot be considered a neutral variable. Because temperature was not controlled, the observed differences cannot be attributed exclusively to species effects.

Lines 382-383: This phrase should go in the discussion section, not here. “In conclusion, BBKV replicated better in the midguts of Aedes species than in those of Culex species”.

Discusion

The discussion does not follow a clear hierarchical structure, for example: (i) main findings, (ii) biological implications, (iii) comparison with previous studies, (iv) limitations, and (v) epidemiological consequences. Instead, topics are interspersed, which disrupts the narrative flow. The discussion does not begin with the most relevant result, such as the efficient infection and dissemination of BBKV in multiple mosquito species or its broad vector range across Aedes and Culex. The paragraphs are weakly connected, and there is no clear guiding thread linking the results to the stated objective of the study. The first paragraph reiterates epidemiological background information (historical isolation, serology, underdiagnosis), which belongs in the Introduction rather than the Discussion. From the second/third paragraph onward, although valid results are discussed, each topic appears almost as an independent block. As a consequence, the main objective of the study (emergence potential) becomes diluted, and the discussion remains largely descriptive rather than interpretative and synthetic.

Reviewer #2: Dear authors,

Thank you for considering my comments and revising the manuscript accordingly.

Reviewer #3: (No Response)

**********

PLOS authors have the option to publish the peer review history of their article (what does this mean?). If published, this will include your full peer review and any attached files.

If you choose “no”, your identity will remain anonymous but your review may still be made public.

**Do you want your identity to be public for this peer review?**  For information about this choice, including consent withdrawal, please see our Privacy Policy.

Reviewer #1: No

Reviewer #2: No

Reviewer #3: No

**Figure resubmission:**

While revising your submission, we strongly recommend that you use PLOS’s NAAS tool (https://ngplosjournals.pagemajik.ai/artanalysis) to test your figure files. NAAS can convert your figure files to the TIFF file type and meet basic requirements (such as print size, resolution), or provide you with a report on issues that do not meet our requirements and that NAAS cannot fix.

After uploading your figures to PLOS’s NAAS tool - https://ngplosjournals.pagemajik.ai/artanalysis, NAAS will process the files provided and display the results in the "Uploaded Files" section of the page as the processing is complete. If the uploaded figures meet our requirements (or NAAS is able to fix the files to meet our requirements), the figure will be marked as "fixed" above. If NAAS is unable to fix the files, a red "failed" label will appear above. When NAAS has confirmed that the figure files meet our requirements, please download the file via the download option, and include these NAAS processed figure files when submitting your revised manuscript.

**Reproducibility:**

To enhance the reproducibility of your results, we recommend that authors of applicable studies deposit laboratory protocols in protocols.io, where a protocol can be assigned its own identifier (DOI) such that it can be cited independently in the future. Additionally, PLOS ONE offers an option to publish peer-reviewed clinical study protocols. Read more information on sharing protocols at https://plos.org/protocols?utm_medium=editorial-email&utm_source=authorletters&utm_campaign=protocols

# Author comment: Author response to Decision Letter 2

**DOI:** 10.1371/journal.pntd.0013051.r004

28 May 2026

Attachment Submitted filename: renamed_fb243.docx

# Peer review: Decision Letter 2

**DOI:** 10.1371/journal.pntd.0013051.r005

**Authors:** Abdallah M. Samy

20 Jul 2026

Dear Dr. Choumet,

We are pleased to inform you that your manuscript 'Vector Competence of Temperate and Tropical Mosquito Species for Babanki virus' has been provisionally accepted for publication in PLOS Neglected Tropical Diseases.

Before your manuscript can be formally accepted you will need to complete some formatting changes, which you will receive in a follow up email. A member of our team will be in touch with a set of requests.

Please note that your manuscript will not be scheduled for publication until you have made the required changes, so a swift response is appreciated.

IMPORTANT: The editorial review process is now complete. PLOS will only permit corrections to spelling, formatting or significant scientific errors from this point onwards. Requests for major changes, or any which affect the scientific understanding of your work, will cause delays to the publication date of your manuscript.

Should you, your institution's press office or the journal office choose to press release your paper, you will automatically be opted out of early publication. We ask that you notify us now if you or your institution is planning to press release the article. All press must be co-ordinated with PLOS.

Thank you again for supporting Open Access publishing; we are looking forward to publishing your work in PLOS Neglected Tropical Diseases.

Best regards,

Abdallah Samy

Section Editor

PLOS Neglected Tropical Diseases

Shaden Kamhawi

co-Editor-in-Chief

PLOS Neglected Tropical Diseases

orcid.org/0000-0003-4304-636XX

Paul Brindley

co-Editor-in-Chief

PLOS Neglected Tropical Diseases

orcid.org/0000-0003-1765-0002

***********************************************************

# Editor report: Acceptance letter

**DOI:** 10.1371/journal.pntd.0013051.r006

**Authors:** Abdallah M. Samy

Dear Dr. Choumet,

We are delighted to inform you that your manuscript, "Vector Competence of Temperate and Tropical Mosquito Species for Babanki virus," has been formally accepted for publication in PLOS Neglected Tropical Diseases.

We have now passed your article onto the PLOS Production Department who will complete the rest of the publication process. All authors will receive a confirmation email upon publication.

The corresponding author will soon be receiving a typeset proof for review, to ensure errors have not been introduced during production. Please review the PDF proof of your manuscript carefully, as this is the last chance to correct any scientific or type-setting errors. Please note that major changes, or those which affect the scientific understanding of the work, will likely cause delays to the publication date of your manuscript. Note: Proofs for Front Matter articles (Editorial, Viewpoint, Symposium, Review, etc...) are generated on a different schedule and may not be made available as quickly.

Soon after your final files are uploaded, the early version of your manuscript will be published online unless you opted out of this process. The date of the early version will be your article's publication date. The final article will be published to the same URL, and all versions of the paper will be accessible to readers.

For Research Articles, you will receive an invoice from PLOS for your publication fee after your manuscript has reached the completed accept phase. If you receive an email requesting payment before acceptance or for any other service, this may be a phishing scheme. Learn how to identify phishing emails and protect your accounts at https://explore.plos.org/phishing.

Thank you again for supporting open-access publishing; we are looking forward to publishing your work in PLOS Neglected Tropical Diseases.

Best regards,

Shaden Kamhawi

co-Editor-in-Chief

PLOS Neglected Tropical Diseases

Paul Brindley

co-Editor-in-Chief

PLOS Neglected Tropical Diseases
